# Estimation of Response Styles Using the Multidimensional Nominal Response Model: A Tutorial and Comparison With Sum Scores

**DOI:** 10.3389/fpsyg.2020.00072

**Published:** 2020-02-06

**Authors:** Carl F. Falk, Unhee Ju

**Affiliations:** ^1^Department of Psychology, McGill University, Montreal, QC, Canada; ^2^Riverside Insights, Itasca, IL, United States

**Keywords:** likert-type items, nominal response model, multidimensional item response theory, response styles, tutorial

## Abstract

Recent years have seen a dramatic increase in item response models for measuring response styles on Likert-type items. These model-based approaches stand in contrast to traditional sum-score-based methods where researchers count the number of times that participants selected certain response options. The multidimensional nominal response model (MNRM) offers a flexible model-based approach that may be intuitive to those familiar with sum score approaches. This paper presents a tutorial on the model along with code for estimating it using three different software packages: flexMIRT®, *mirt*, and M*plus*. We focus on specification and interpretation of response functions. In addition, we provide analytical details on how sum score to scale score conversion can be done with the MNRM. In the context of a real data example, three different scoring approaches are then compared. This example illustrates how sum-score-based approaches can sometimes yield scores that are confounded with substantive content. We expect that the current paper will facilitate further investigations as to whether different substantive conclusions are reached under alternative approaches to measuring response styles.

## Introduction

Likert-type items are ubiquitous throughout the social sciences. Some example uses of such items include measurement of positive and negative affect (Watson et al., 1988), and personality traits such as the Big Five (Goldberg, 1992) and self-esteem (Tafarodi and Swann, 2001). Despite the popularity of Likert-type items, one critique focuses on the vulnerability of such items to *response styles*, or peculiarity in how respondents use response options that are not relevant to item content, including extreme responding (ERS; selection of the lowest or highest anchor), midpoint responding (MRS; use of the middle anchor), acquiescence (ARS; agreement to the items), and so on (Baumgartner and Steenkamp, 2001). Although the nature of response styles has been debated for many decades (Cronbach, 1950; Couch and Keniston, 1961; Hamilton, 1968), recent research suggests that response styles may be individual characteristics that have some level of stability over time (Wetzel et al., 2016) and are consistent both within and across surveys or items from different substantive traits (Javaras and Ripley, 2007; Wetzel et al., 2013). Yet, response styles may be sensitive to the format of the Likert scale (Diamantopoulos et al., 2006; Weijters et al., 2010). Also, when response styles occur, they can reduce the validity of measurements, possibly inducing illusory correlations among variables, and distortion of mean differences across groups (Baumgartner and Steenkamp, 2001; De Jong et al., 2008; Bolt and Johnson, 2009; Buckley, 2009).

However, many of the most cited findings regarding response styles' demographic and personality correlates, cross-cultural variability, and temporal stability have used a *sum-score-based* approach to measurement (Hui and Triandis, 1989; Chen et al., 1995; Baumgartner and Steenkamp, 2001; van Herk et al., 2004; Johnson et al., 2005; Harzing, 2006). Specifically, a researcher may simply compute the sum or mean number of times a participant selected the endpoint categories to establish a measure of ERS. Although easy to implement, this approach is not based on an explicit measurement model and it is unclear under what conditions it can sufficiently disentangle style from content (e.g., see De Beuckelaer et al., 2010). Furthermore, conclusions regarding the consequences of response styles may depend on the methodology employed to measure them.

Alternatively, item response models can measure response styles, and include, but are not limited to multiprocess models (e.g., Thissen-Roe and Thissen, 2013; Khorramdel and von Davier, 2014; Plieninger and Meiser, 2014; Böckenholt and Meiser, 2017), unfolding models (Liu and Wang, 2019), and the multidimensional nominal response model (MNRM; e.g., Bolt and Newton, 2011; Kieruj and Moors, 2013; Falk and Cai, 2016). Such approaches arguably rest upon testable assumptions and can handle some situations that sum scores cannot (e.g., planned missing data designs), and have numerous other advantages (e.g., conditional standard errors for score estimates). We argue that use of the MNRM can be similar to researchers' intuitions regarding sum scores and provides a suitable alternative. To elaborate, suppose for ERS we assign a score of “1” to the endpoint categories and “0” to intermediate categories when determining how to create a sum score. These same 0 or 1 values could be used to specify the *scoring functions* of the MNRM, which determine how the order of categories for each item relate to the latent traits. This strategy has recently appeared in the methodology literature with item slopes, or “loadings,” being fixed (Bolt and Newton, 2011) or varying across both style and substantive traits (Falk and Cai, 2016). Thus, it is possible to study whether some items are better for measuring a particular construct, and whether certain content, item stems, or response options are more likely to yield response styles (Deng and Bolt, 2017). The MNRM can also be used when a style, such as socially desirable responding (SDR), is defined in a different way across items (Kuncel and Tellegen, 2009). And recent work illustrates the utility of the MNRM in investigating the effects of response styles on the test construction process and measurement precision of model-based scores (Adams et al., 2019).

Despite the connection between these variants of the MNRM and sum scores, comparisons are rare, and further study of response styles with the MNRM depends on the availability of illustrative examples. One goal of this paper is to provide a tutorial along with code for estimating the MNRM for response styles. Thus, although tutorials are available for multiprocess models (Böckenholt and Meiser, 2017), none are apparently available for the MNRM. Schneider (2018) provided code for M*plus* (Muthén and Muthén, 1998-2017), yet for a more constrained model analogous to that by Bolt and Newton (2011). Falk and Cai (2016) provided R code in their Supplementary Materials, yet their example was slow to estimate and not easily adaptable to other measurement instruments. Anecdotally, their presentation of the MNRM may be challenging to understand; here we draw explicit connections between the MNRM and logistic regression, and with existing sum-score-based approaches. The MNRM is now available within flexMIRT® (Cai, 2017) and *mirt* (Chalmers, 2012), and we provide code in Supplementary Materials to estimate Falk and Cai's (2016) approach with these programs and with M*plus*.

As context, it has been argued that measurement of response styles is best done using a set of “heterogeneous” items (Greenleaf, 1992; De Beuckelaer et al., 2010). We understand *heterogeneity* to refer to content, in that items used to assess response style should come from measurement instruments meant to assess different domains and have low inter-item correlations. Items may be drawn from standard inventories (Weijters, 2006) or from vignettes (e.g., Bolt et al., 2014; Baird et al., 2017). Such items, however, are not devoid of content, pairs of items may be correlated, and individuals may respond in an idiosyncratic way to some items. In contrast, the MNRM can be fit to data intended to measure just a single substantive construct, raising the question of how much content heterogeneity is necessary. We provide an empirical example in which many items with many response options measure a single substantive construct, along with an illustration of the separation of style vs. content scores for the MNRM and sum scores.

Finally, once the MNRM is estimated, there are several major ways of estimating scores for the substantive and style traits (often called *scale scores*), some of which require knowledge of the full response pattern for each participant. Alternatively, sometimes it is easier to generate an approximate scale score through use of sum score to scale score translation tables (Thissen et al., 2001). This scoring approach requires the researcher to only compute a sum score and then use a table to find an approximate corresponding scale score for the latent trait; the full response pattern is not required. Our example provides an additional comparison with the use of sum score to scale score conversion for MNRM response style models. In what follows, we present notation, a dataset, and details for computing sum scores and estimating the MNRM. We focus heavily on interpretation of the MNRM, anticipate common questions regarding definition of scoring functions, touch on use of software, as well as briefly discuss model fit. We then compare sum scores vs. two scoring procedures for the MNRM.

## Empirical Illustration

### Notation and Data

To introduce notation, suppose that *i* = 1, 2, …, *N* people respond to *j* = 1, 2, …, *n* items. Person *i'*s observed response to item *j* is denoted *y*_*ij*_. For measuring a substantive trait, the response options are often coded in an ordinal fashion with *k* = 0, …, *K*_*j*_ − 1 indexing the categories for item *j*. For convenience, additional commonly used symbols appear in Table 1 and will be discussed in further detail as they appear. To reduce notational clutter, we will omit item and person subscripts as often as possible. However, inspection of Table 1 reveals that the number of categories, slopes, intercepts, and scoring functions may vary across items; latent traits or sum score composites may vary across people; and observed responses (original or recoded) vary across both people and items. Finally, any vectors or matrices in our paper will appear in bold.

**Table 1:** Some important symbols used in this manuscript.

**Symbol**	**Purpose**	**Notation omitting person and/or item subscript**
*y*_*ij*_	Person *i*'s observed response to item *j*.	*y* or *y*_*j*_
*t*_*ij*_	Recoded response for person *i* and item *j*.	*t* or *t*_*j*_
*K*_*j*_	Total number of categories for item *j*.	*K*
*v*_*i,d*_	Person *i's* sum score composite for construct *d*.	*v*_*d*_
*x*_*i,d*_	Person *i's* score on the latent trait for construct *d*.	*x*_*d*_
*a*_*j,d*_	Slope (or loading) for item *j* and construct *d*.	*a*_*d*_
*c*_*j,k*_	Intercept for item *j* and category *k*.	*c*_*k*_
*s*_*j,kd*_	Scoring function value for item *j*, category k and construct *d*.	*s*_*kd*_

To make this example concrete, consider *N* = 586 participants that completed *n* = 35 items measuring quality of life (QOL) on a 7-point Likert scale (*K*_*j*_ = 7 for all *j*). This dataset was published by Lehman (1988) and is included in online examples for flexMIRT®. All items are coded such that higher scores indicate higher QOL. Assuming the *n* items are intended to measure a single underlying substantive construct, a sum score composite for QOL is computed by adding up scores for the items:

(1) $$\begin{array}{l} v_{QOL}=\;y_{1}+y_{2}+\cdots +y_{n}=\sum_{j=1}^{n}y_{j} \end{array}$$

Sum scores also correlate perfectly with taking the *mean* of all responses as an index of QOL.

To understand sum-score-based approaches to response styles, consider recoding the original categories. For assessing ERS as defined by responding to the lowest and highest categories, we use *y*_*j*_ to create *t*_*j*_ using the following mapping: {0, 1, 2, 3, 4, 5, 6} ↦ {1, 0, 0, 0, 0, 0, 1}. Specifically, when creating *t*_*j*_, we may change all “0” and “6” responses to “1” and all other responses to “0.” A sum-score-based measure of ERS is then the composite:

(2) $$\begin{array}{l} v_{ERS}=\;t_{1}+t_{2}+\cdots +t_{n}=\overset{n}{\underset{j=1}{\sum }}t_{j} \end{array}$$

Responses from several participants for all 35 items, their recoded response patterns, and their sum-score-based composites appear in Table 2. Further details of this table will be discussed later.

**Table 2:** Example response patterns, sum scores, and EAP scores.

		**QOL Scores**		
**Subject**	**Original response pattern**	**v_QOL_**	**SS EAP_QOL_**	**EAP_QOL_**
1	11111131116361131163661661161363466	105	−0.74	−0.64
2	00000600043324433564443564551452235	105	−0.74	−0.66
3	51112454246566624524556563466652344	145	0.40	0.05
4	66466555511131144644434660556555445	145	0.40	0.20
5	36644444244455221553545332566546556	147	0.47	0.28
6	63333444455555555555545665002055555	147	0.47	0.46
		**ERS Scores**		
**Subject**	**Recoded ERS response pattern**	**v**_**ERS**_	**SS EAP**_**ERS**_	**EAP**_**ERS**_
1	00000000001010000010110110010010011	11	0.56	0.96
2	11111111100000000010000010000000000	11	0.56	0.80
3	00000000001011100000001010011100000	9	0.38	0.60
4	11011000000000000100000111001000000	9	0.38	0.41
5	01100000000000000000000000011001001	6	0.05	0.15
6	10000000000000000000000110110100000	6	0.05	0.16
		**MRS Scores**		
**Subject**	**Recoded MRS response pattern**	**v**_**MRS**_	**EAP**_**MRS**_	**EAP**_**MRS**_
1	00000010000100010001000000000101000	6	0.42	0.44
2	00000000001100011000001000000000010	6	0.42	0.21
3	00000000000000000000000001000000100	2	−0.38	−0.58
4	00000000000010000000010000000000000	2	−0.38	−0.54
5	10000000000000000001000110000000000	4	0.07	−0.04
6	01111000000000000000000000000000000	4	0.07	0.37

*Dimension subscripts are as follows: QOL, quality of life; ERS, extreme response style; MRS, midpoint response style*.

In either example above, each item is given *equal weight* when computing a sum score. For instance, item 2 will contribute the same as item 1 to a QOL score, even if item 1 is more closely related to QOL. Since the unique properties of each item are not considered, it is also difficult to tell, for example, if the reason a participant selected “6” for an item is because they are high on the substantive construct, high on ERS, or the item is just easy to endorse.

To later connect the MNRM with sum scores, suppose we represent how the items were recoded in a vector called a *scoring function*. The scoring function for construct *d* for a particular item is denoted **s**_d_ = [*s*_1*d*_ *s*_2*d*_ ⋯ *s*_*Kd*_] and has as many elements as there are categories. For this example, **s**_QOL_ = [0 1 2 3 4 5 6] represents QOL whereas **s**_ERS_ = [1 0 0 0 0 0 1] represents ERS. In other words, the scoring functions determine how an item's categories are related to a construct, and one application involves how to (re-)code the original categories when computing sum scores. The MNRM will use such scoring functions, but models both substantive and response style constructs simultaneously and can consider the properties of test items.

### Multidimensional Nominal Response Model (MNRM)

#### Model Representation and Interpretation

The MNRM is based in part on a unidimensional model by Bock (1972). Recent work provides additional insight into the interpretation and history of the MNRM (Thissen et al., 2010; Thissen and Cai, 2016). We argue that knowledge of logistic regression is sufficient for understanding the MNRM, and we assume such knowledge in what follows.

**Figure 1:**
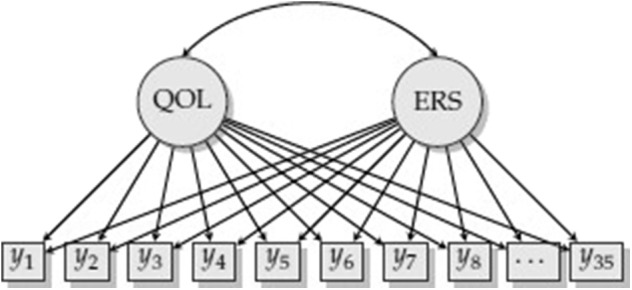
Model with quality of life (QOL) and extreme response style (ERS).

Consider measurement of QOL and ERS (Figure 1). Each item response is regressed on QOL and ERS, which are correlated. More formally, the predictor variables are a participant's scores on *d* = 1, 2, …, *D* latent traits, $\text{x}={\left[x_{1}\;x_{2}\;\cdots \;x_{D}\right]}^{T}$, and the outcomes are responses to particular items. If this were a confirmatory factor analysis (CFA) model, the relationship between the latent traits and each of the items would resemble a linear regression. For example, item *j* would be regressed on both QOL and ERS, *y*_*j*_ = ι_*j*_ + λ_*j,QOL*_*x*_*QOL*_ + λ_*j,ERS*_*x*_*ERS*_ + ε_*j*_ where ι_*j*_ is an intercept, λ_*j,QOL*_ and λ_*j,ERS*_ are loadings (or slopes) for these latent dimensions, and ε_*j*_ is an error term. The CFA framework, however, does not make sense. Aside from such a model not being identified (QOL and ERS are redundant since all items load on both factors), we would also not expect a linear relationship between ERS and item responses. Using the MNRM, we can decide the type of relationship between ERS and the item responses, while specifying a *different* type of relationship for QOL. If such a constrained version of the MNRM is used, the model in Figure 1 becomes identified and makes more substantive sense.

One way of representing the MNRM is that of a series of logistic regressions. Suppose that we know that a response is in one of two categories: *k* or *k*′. Under this setup, we define $T_{k,k^{\prime }}^{*}$ as the probability that the response is category *k*, and $T_{k^{\prime },k}^{*}$ as the probability that the response is *k*′ (note the different subscripts). Since we are currently considering only two categories, these two probabilities must sum to one: ${T_{k,k^{\prime }}^{*}+T}_{k^{\prime },k}^{*}=1$. A logistic regression under the MNRM can then be represented as the log-odds of choosing category *k* instead of category *k*′:

(3) $$\begin{array}{l} \text{log}\frac{T_{\;k,k\prime }^{*}}{T_{\;k\prime ,k}^{*}}=c_{\;k,k\prime }^{*}+a_{1}\;(s_{k\prime 1}-s_{\mathrm{k\prime}1})\;x_{1}\\ \;+\cdots +a_{D}(s_{kD}-s_{k\prime D})\;x_{D} \end{array}$$

To unpack the right-hand side of Equation (3), note that $c_{k,k^{\prime }}^{*}=c_{k}-c_{k^{\prime }}$ is an intercept with *c*_*k*_ and $c_{k^{\prime }}$ as category-specific intercepts, but are not of central importance to us at the present. Next, *a*_*d*_ is a slope for dimension *d* that represents the strength of relationship between the construct and response. Thus, *a*_*d*_ is analogous to a factor loading, and conceptually similar to a regression coefficient in a logistic regression. Finally, *s*_*kd*_ is a *scoring function* value for category *k* and dimension *d*. Note the re-use of notation in that the scoring function values, *s*_*kd*_, are the same as those used for sum scores in the previous section. Returning to measurement of just QOL and ERS, Equation (3) becomes the following:

(4) $$\begin{array}{l} \text{log}\frac{T_{\;k,k\prime }^{*}}{T_{\;k\prime ,k}^{*}}=c_{\;k,k\prime }^{*}+a_{QOL}\;(s_{k,QOL}-s_{k\prime ,QOL})\;x_{QOL}\\ \;+a_{ERS}(s_{k,ERS}-s_{k\prime ,ERS})\;x_{ERS} \end{array}$$

QOL and ERS have slopes, *a*_*QOL*_ and *a*_*ERS*_, respectively, and there is some difference in the scoring function values that also determines whether *x*_*QOL*_ or *x*_*ERS*_ is related to a choice between *k* and *k*′. Further understanding can be obtained by examining specific categories, using **s**_QOL_ = [0 1 2 3 4 5 6], and **s**_ERS_ = [1 0 0 0 0 0 1], and example item parameters: *a*_*QOL*_ = 0.46, *a*_*ERS*_ = 1.03, *c*_0_ = 0.00, *c*_1_ = 1.16, *c*_2_ = 1.17, *c*_3_ = 2.56, *c*_4_ = 2.89, *c*_5_ = 2.96, *c*_6_ = 2.72. If we compare the second (*k* = 1) and first (*k*′ = 0) categories, the expression in (4) simplifies:

(5) $$\begin{array}{r} \log\frac{T_{1,0}^{*}}{T_{0,1}^{*}}=c_{1,0}^{*}+a_{QOL}\;\left(s_{1,QOL}-s_{0,QOL}\right)\;x_{QOL}\\ +a_{ERS}\;\left(s_{1,ERS}-s_{0,ERS}\right)\;x_{ERS}\\ \;=c_{1,0}^{*}+a_{QOL}\;\left(1-0\right)\;x_{QOL}+a_{ERS}\;\left(0-1\right)\;x_{ERS}\\ \;=\left(c_{1}-c_{0}\right)+a_{QOL}x_{QOL}-a_{ERS}x_{ERS}\\ =1.16+0.46x_{QOL}-1.03x_{ERS} \end{array}$$

Assuming *a*_*QOL*_ and *a*_*ERS*_ are both positive, choice of *k* = 1 (vs. *k*′ = 0) is positively related to the QOL dimension (*a*_*QOL*_*x*_*QOL*_ or 0.46*x*_*QOL*_) but negatively related to ERS (−*a*_*ERS*_*x*_*ERS*_ or −1.03*x*_*ERS*_). That is, higher QOL results in a choice of this higher category (consistent with higher scores indicating higher QOL), but higher ERS may lead someone to be less likely to endorse *k* = 1 since it is not an endpoint category, but *k*′ = 0 is. We can also say that for a 1-unit increase in *x*_*QOL*_, there is a *a*_*QOL*_ change in the log-odds of choosing category 1 instead of category 0.

Consider another example comparing categories 0 and 2:

(6) $$\begin{array}{l} \log\frac{T_{2,0}^{*}}{T_{0,2}^{*}}=c_{2,0}^{*}+a_{QOL}\;\left(2-0\right)\;x_{QOL}+a_{ERS}\;\left(0-1\;\right)\;x_{ERS}\\ \;=\left(c_{2}-c_{0}\right)+{2a}_{QOL}x_{QOL}-a_{ERS}x_{ERS}\\ \;=1.17+2\left(0.46\right)x_{QOL}-1.03x_{ERS} \end{array}$$

Here choice of *k* = 2 vs. *k*′ = 0 is more indicative of QOL (by 2*a*_*QOL*_ = 2 × 0.46) since *k* = 2 is an even higher category than *k* = 1 from the previous example. ERS retains the same negative relationship with this pair of categories because *k*′ = 0 is an endpoint category, but *k* = 2 is not.

Finally, consider the following:

(7) $$\begin{array}{l} \log\frac{T_{2,1}^{*}}{T_{1,2}^{*}}=c_{2,1}^{*}+a_{QOL}\;\left(2-1\right)\;x_{QOL}+a_{ERS}\;\left(0-0\;\right)\;x_{ERS}\\ \;=\left(c_{2}-c_{1}\right)+a_{QOL}x_{QOL}\\ \;=0.01+0.46x_{QOL} \end{array}$$

Here, the log-odds of choosing *k* = 2 vs. *k*′ = 1 is related to QOL by just *a*_*QOL*_, since *k* = 2 is only one category higher than *k*′ = 1. ERS is apparently unrelated to a choice between these two categories as neither is an endpoint category. Similarly, this same relationship with ERS is also apparent for the choice of *k*′ = 0 vs. *k* = 6 as *both* are endpoint categories.

The pairs of logistic regressions in the above examples are estimated simultaneously, and another way to represent the MNRM proposed by Thissen and Cai (2016) is as follows:

(8) $$\begin{array}{l} T\left(k|\text{x}\right)\\ =\frac{\exp\left(a_{1}s_{k1}x_{1}+a_{2}s_{k2}x_{2}+\ldots +a_{D}s_{kD}x_{D}+c_{k}\right)}{\sum_{m}^{K-1}\exp\left(a_{1}s_{m1}x_{1}+a_{2}s_{m2}x_{2}+\ldots +a_{D}s_{mD}x_{D}+c_{m}\right)} \end{array}$$

This parameterization matches that used by flexMIRT® (Cai, 2017) and the constrained version of the MNRM used by *mirt* (Chalmers, 2012). Equation (8) traces the probability that a response will be in category *k* at different levels of the latent traits. If we consider the above example item parameters, we can plug in particular values of the latent traits and examine the resulting probabilities computed by *T*(*k*|**x**). We encourage verifying understanding of Equation (8) by replicating the values in Table 3. For example, when a participant is low (*x*_*QOL*_ = −3) on QOL, there is only a 0.03 proportion of the time we would expect them to select the lowest category (*k* = 0) when ERS is also low (*x*_*ERS*_ = −3), but a 0.94 proportion when ERS is high (*x*_*ERS*_ = 3).

**Table 3:** Probability of response in each category at several values of the latent traits.

**Latent Traits**		**Probability of Response**, **T**(**k**|**x**)						
**x**_QOL_	**x**_ERS_	***0***	***1***	***2***	***3***	***4***	***5***	***6***
−3	−3	0.03	0.60	0.15	0.15	0.05	0.01	0.00
0	−3	0.00	0.06	0.06	0.22	0.31	0.34	0.01
3	−3	0.00	0.00	0.00	0.03	0.16	0.70	0.10
−3	3	0.94	0.03	0.01	0.01	0.00	0.00	0.00
0	3	0.05	0.01	0.01	0.03	0.04	0.05	0.81
3	3	0.00	0.00	0.00	0.00	0.00	0.01	0.98

**Figure 2:**
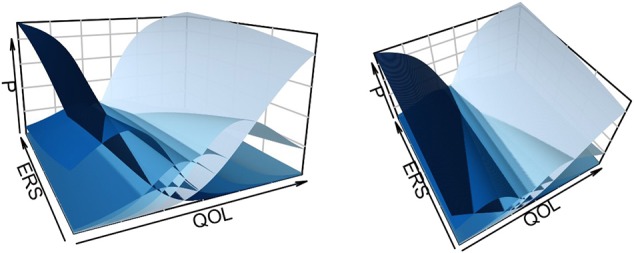
Category response functions for Item 2 from a model with QOL and ERS.

If we construct a two-dimensional grid along QOL and ERS, we can then plot the probability of selecting each category (the *z*-axis “P”) in three-dimensional space (Figure 2). Here QOL ranges from −6 to 6 and ERS from −1 to 1, and darker shades of blue indicate lower categories. As ERS increases, the endpoint categories become more dominant response options—participants are more likely to pick such categories even if they are not very high or low on QOL. The intermediate categories (e.g., *k* = 1 and *k* = 5), become more dominant response options as ERS decreases (For two-dimensional slices of such plots, see Falk and Cai, 2016).

#### Choice of Scoring Functions

Researchers have much flexibility in choosing scoring functions. We have focused on ERS, but only because it is so common in the literature. For instance, if SDR is defined by selection of a particular category, or if the social desirability of all categories for an item is evaluated by an independent set of raters, then it may also be possible to define scoring functions such that higher values correspond to higher SDR. Falk and Cai (2016, p. 334, Table 2; see also Wetzel and Carstensen, 2017) provide this and additional examples for a 5-category item, noting that “A useful heuristic is to consider scoring functions analogous to contrasts used for categorical predictors in linear models (e.g., regression, analysis of variance)” (p. 331). To provide other examples, it may be possible to conceptualize a tendency to stay close to the middle of the response scale with a scoring function such as **s** = [0 0 1 1 1 0 0] or by defining MRS as exclusive use of the middle category, **s** = [0 0 0 1 0 0 0]. The definition of **s** may therefore depend heavily on substantive theory and a certain extent on model fit or some other criterion. The flexibility of the MNRM in this case is both a blessing and curse. However, we argue that substantive researchers who use sum scores are already making similar assumptions about scoring functions.

Part of the above analogy to contrast coding in linear models refers to linear dependence among the scoring functions. The model in Figure 1 is estimable because QOL and ERS have different scoring functions. We cannot add a third construct with a scoring function of [0 1 1 1 1 1 0] as this would be redundant with our definition of ERS. If there are few categories, say 4, then modeling acquiescence may be controversial with **s** = [0 0 1 1] as this may be too similar to **s** for a substantive trait. Thus, this advice is similar to the choice of categorical codes in linear regression to avoid redundancy and multicollinearity among predictors, else risk estimation problems. Even if a model is identified, parameter estimates may be difficult to find if there are not enough good items and participants. An *ad-hoc* way to check for identification is to begin model estimation again with different starting values for parameters. Convergence to a different solution provides evidence that the model may not be identified.

In many cases, the most interpretability for scoring functions may be offered by using integer values starting with 0 as a reference category on one end of the latent continuum and higher integer values representing responses toward the other end of the continuum. For instance, if we instead used [1 − 1 − 1 − 1 − 1 − 1 1] for assessing ERS, Equations (5) and (6) would contain terms such as −2ǎ_*ERS*_*x*_*ERS*_ instead of −*a*_*ERS*_*x*_*ERS*_ where ǎ_*ERS*_ = 0.5*a*_*ERS*_. While this is an equivalent model (−2ǎ_*ERS*_ = −*a*_*ERS*_), the scaling of the slope changes to compensate for changes in the scoring functions, and software would yield ǎ_*ERS*_ = 0.515 as output instead of *a*_*ERS*_ = 1.03. Use of 1 and 0 instead for ERS makes such slopes analogous to those obtained from a logistic regression where the ERS factor is standardized (since the model is usually identified by fixing the variance of the ERS factor to one). That is, a one standard deviation change in ERS corresponds to a *a*_*ERS*_ change in the log-odds of obtaining an observed response for an endpoint vs. non-endpoint category. Convention dictates that scoring function values used for QOL, [0 1 2 3 4 5 6], are the same as those used by the generalized partial credit model (GPC; Muraki, 1992).

A limitation is that it may not be easy to directly compare item slopes from dimensions that have different scoring functions. For example, it may be tempting to say that ERS is more strongly related to the item than is QOL since *a*_*ERS*_ = 1.03, but *a*_*QOL*_ = 0.46. Such an interpretation is imprecise as this ignores the fact that the item has seven categories for measuring QOL, but essentially only two for ERS due to its scoring function definition. Thus, the item still may provide more information regarding the participant's standing on QOL than it does for ERS, and additional work may still be required to further examine item information in the context of such a multidimensional model. It may instead be more worthwhile to compare slopes across items for a single dimension, such as comparing which items are most closely related to ERS (e.g., Deng and Bolt, 2017; Ju and Falk, 2019).

### Model Estimation and Software

The models we report were estimated using maximum marginal likelihood with the Expectation-Maximization algorithm (EM-MML; Bock and Aitkin, 1981), though other algorithms are a good option when there are more than two or three latent traits (Cai, 2010). To estimate the model in Figure 1, scoring functions must be specified such that one dimension represents QOL and the other ERS. The various software programs accomplish this in different ways, and we further elaborate on some of these details in Supplementary Materials.

#### flexMIRT®

It is possible to tell flexMIRT® to fix the scoring functions of the MNRM to prespecified values. These values appear in the first column of a *K* × (*K* − 1) matrix, **T**_a,d_. The subscript *a* indicates that this matrix is relevant for slopes, and *d* specifies a latent dimension. For example, for QOL and a 7-category item, this is a 7 × 6 matrix and corresponds to the following:^1^

$$\begin{array}{l} {\text{T}}_{\text{a},\text{QOL}}=\left[\begin{array}{cccccc} 0 & 0 & 0 & 0 & 0 & 0\\ 1 & 0 & 0 & 0 & 0 & 0\\ 2 & 0 & 0 & 0 & 0 & 0\\ 3 & 0 & 0 & 0 & 0 & 0\\ 4 & 0 & 0 & 0 & 0 & 0\\ 5 & 0 & 0 & 0 & 0 & 0\\ 6 & 0 & 0 & 0 & 0 & 0 \end{array}\right] \end{array}$$

If we instead wish to model ERS with a scoring function of **s**_**ERS**_ = [1 0 0 0 0 0 1], we may specify different values for the first column of the relevant matrix:

$$\begin{array}{l} {\text{T}}_{\text{a},\text{ERS}}=\left[\begin{array}{cccccc} 1 & 0 & 0 & 0 & 0 & 0\\ 0 & 0 & 0 & 0 & 0 & 0\\ 0 & 0 & 0 & 0 & 0 & 0\\ 0 & 0 & 0 & 0 & 0 & 0\\ 0 & 0 & 0 & 0 & 0 & 0\\ 0 & 0 & 0 & 0 & 0 & 0\\ 1 & 0 & 0 & 0 & 0 & 0 \end{array}\right] \end{array}$$

To use these matrices as input to flexMIRT®, we use what resembles a block-diagonal super-matrix:

$$\begin{array}{l} \;\left[\begin{array}{cc} {\text{T}}_{a,QOL} & 0\\ 0 & {\text{T}}_{a,ERS} \end{array}\right] \end{array}$$

where **0** is a matrix of zeros of appropriate dimensions. An example of such a matrix is found in the control file “QOL_Calib_1dimERS.txt,” and annotated control files further elaborate on exactly how to input this matrix. If additional dimensions are required, the super-matrix provided as input can be expanded. For instance, if we add a third factor, MRS, with scoring function **s**_MRS_ = [0 0 0 1 0 0 0], this is accomplished in the “QOL_Calib_1dimERSMRS.txt” file.

#### mirt

For the *mirt* package in R (see “mirtcode.R”), the models discussed here are specified such that all items load on all dimensions and the GPC model is chosen as the item type for all items. Custom scoring functions for each dimension are input using a special argument, “gpcm_mats.” This argument takes a list of matrices, each corresponding to a *K*_*j*_ × *D* matrix. For a model with QOL, ERS, and MRS, this matrix may resemble the following for a 7-category item:

$$\begin{array}{l} \left[\begin{array}{ccc} 0 & 1 & 0\\ 1 & 0 & 0\\ 2 & 0 & 0\\ 3 & 0 & 1\\ 4 & 0 & 0\\ 5 & 0 & 0\\ 6 & 1 & 0 \end{array}\right] \end{array}$$

Thus, each column corresponds to the scoring function for a particular latent dimension. Here the QOL, ERS, and MRS scoring functions are in the first, second, and third columns, respectively.

#### M*plus*

M*plus* uses a multidimensional version of Bock's (1972) nominal categories model:

(9) $$\begin{array}{l} T\left(k|\text{x}\right)\\ =\frac{\exp\left({\tilde{a}}_{k1}x_{1}+{\tilde{a}}_{k2}x_{2}+\ldots {+\tilde{a}}_{kD}x_{D}+c_{k}\right)}{\sum_{m}^{K-1}\exp\left({\tilde{a}}_{m1}x_{1}+{\tilde{a}}_{m2}x_{2}+\ldots {+\tilde{a}}_{mD}x_{D}+c_{m}\right)} \end{array}$$

where ã_*kd*_ is a slope for category *k* and dimension *d*^2^. Each ã_*kd*_ is a slope that represents change in the log-odds of choosing category *k* over some reference category, *k*′ (due to a 1 unit change in *x*_*d*_). Thus, these slopes have a familiar logistic regression interpretation. To see the connection between this and Equation (8), notice that category slopes are equivalent to the product of an overall slope for dimension *d* and scoring function value, ã_*kd*_ = *a*_*d*_*s*_*kd*_. Although it is typical to use the first category as the reference category (i.e., ã_0*d*_ = 0 for all *d* and *c*_0_ = 0), M*plus* uses the *last* category and sets its slope and intercept to zero (ã_(*K* − 1)*d*_ = 0 for all *d* and *c*_(*K* − 1)_ = 0) − *a* default that cannot be changed. Thus, standard M*plus* output is analogous to setting the last scoring function value for each dimension, *s*_(*K* − 1)*d*_, to zero, which may not be congruent with all scoring functions of interest. Use of M*plus* therefore requires additional work to obtain the desired scoring functions and overall item slopes (e.g., Huggins-Manley and Algina, 2015; Schneider, 2018).

To obtain the GPC model for measuring QOL, a strategy employed by Huggins-Manley and Algina (2015) starts by reverse coding the items, resulting in the original first category now coded as the last category (its scoring function value and intercept fixed to zero). It may then be easiest to consider reversing the order of all scoring function values we have previously presented in this manuscript. For QOL, we can use equality constraints to obtain a scoring function that is reversed and with a zero in its last position: **s** = [6 5 4 3 2 1 0]. Determining how to impose constraints may be easiest if considering a category slope with 1 (or −1) as its corresponding scoring function value, and imposing constraints relative to that slope. In this case, ã_5,*QOL*_ = *a*_*QOL*_*s*_5,*QOL*_ = *a*_*QOL*_(1) = *a*_*QOL*_, or that this category slope is equivalent to the overall slope for this item on the QOL dimension. Next, consider ã_4,*QOL*_ = *a*_*QOL*_*s*_4,*QOL*_ = *a*_*QOL*_(2) = 2*a*_*QOL*_. After a little algebra, we see that, ã_4,*QOL*_ = 2ã_5,*QOL*_, and this constraint can be implemented in the CONSTRAINT section of the M*plus* control file. In addition, ã_3,*QOL*_ = 3ã_5,*QOL*_, ã_2,*QOL*_ = 4ã_5,*QOL*_, and so on. In brief, (*K* − 1) constraints per dimension are typically required for each item.

For measuring MRS, zero is in the last position for the scoring function, regardless of whether the categories are in reverse order: **s** = [0 0 0 1 0 0 0]. Thus, all other category slopes for MRS (except for the middle category) may be fixed to zero. Recognizing that the last scoring function value must always be zero, for ERS we may instead subtract 1 from the scoring function values and use: **s** = [0 −1 −1 −1 −1 −1 0]. The category slopes for the five middle categories can be set equal, and the category slope in the first position fixed to zero. Note however, that any of the middle category slopes are equivalent to −*a*_*ERS*_, for example, ã_3,*ERS*_ = *a*_*ERS*_*s*_3,*ERS*_ = *a*_*ERS*_(−1) = −*a*_*ERS*_. The MNRM behaves similar rules as other factor analytic models in that some factors may be reflected (i.e., higher values on the latent variable may mean lower scores on the construct, depending on whether loadings are positive or negative). To avoid reflection for ERS, we use negative starting values for the middle category slopes. Finally, remember that intercepts may appear in the opposite order in the output, and it may be desirable to convert one of the category slopes to the overall slope, *a*_*d*_, for each dimension. Re-ordering of intercepts and this slope conversion can be facilitated by defining new parameters in the CONSTRAINT section of the M*plus* control file.

### Results of Fitted Models

A summary of fit for models with all combinations of ERS and MRS (one style dimension, both, or neither) appears in Table 4, based on output from flexMIRT®. The limited information fit statistic, *C*_2_, was used based on Cai and Monroe (2014), along with RMSEA (e.g., Maydeu-Olivares and Joe, 2014) and a Tucker-Lewis Index (TLI; Cai and Monroe, 2013). Inspecting AIC and BIC, the model with both ERS and MRS fit best as these values are at their lowest. If one prefers, nested models could be compared with a likelihood ratio test (but see Maydeu-Olivares and Cai, 2006). For example, the QOL only model can be obtained from any of the other models by fixing all response style slopes to zero and not estimating correlations between QOL and other dimensions. The likelihood ratio tests for comparing this model to that with both ERS and MRS proceeds by taking the difference in −2 times the log-likelihood (−2*LL*) from these two models (67424–63958) and comparing to a central chi-square distribution with degrees of freedom equal to difference in the number of free parameters (318–245). In this case, the model with both ERS and MRS fits better, χ^2^(73) = 3466, *p* < 0.001. It would not be possible, however, using this approach to compare the models containing only ERS or MRS, as these models are not nested.

**Table 4:** Summary of model fit for quality of life data.

**Model**	**C_2_**	***df***	***p***	**RMSEA**	**TLI**	**AIC**	**BIC**	**−2LL**	***np***
QOL	5,266	560	<0.01	0.12	0.85	67,914	68,985	67,424	245
QOL, MRS	4,158	524	<0.01	0.11	0.88	66,887	68,116	66,325	281
QOL, ERS	3,694	524	<0.01	0.10	0.89	65,570	66,799	65,008	281
QOL, ERS, MRS	2,761	487	<0.01	0.09	0.92	64,594	65,984	63,958	318

*QOL = quality of life; ERS = extreme response style; MRS = midpoint response style; np = number of estimated parameters*.

We will present scoring results based on the ERS and MRS model, yet none of the models had stellar fit (e.g., *C*_2_ rejects all models, and associated fit indices look mixed). However, guidelines for RMSEA and TLI in this context have yet to be developed and may not be comparable to their counterpart in structural equation modeling.

**Figure 3:**
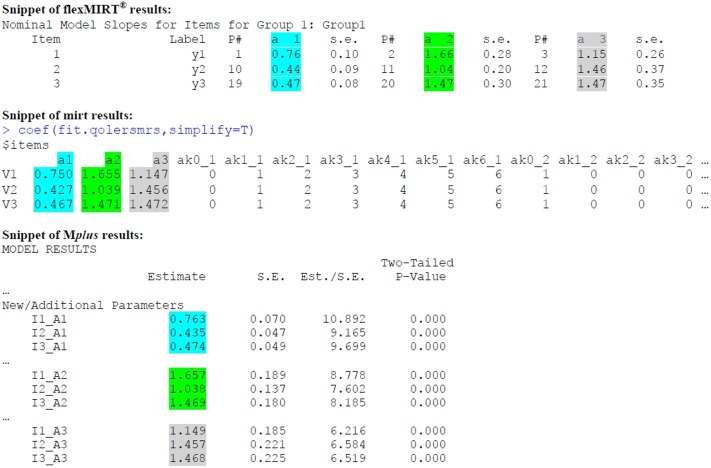
Slope estimate output for the first three items.

As item parameters are used in scoring, it may be useful to also inspect estimates before scoring results. Full output from flexMIRT® and M*plus* appears in Supplementary Materials, and the provided code for *mirt* can also be used to obtain item parameter estimates. In Figure 3, we provide short snippets of output from all three programs so that the reader may more easily identify slope estimates from the MNRM. Highlighted are slope estimates for QOL (cyan), ERS (green), and MRS (gray). In general, slope estimates will be nearly the same across programs, yet some small discrepancies can be seen at the second decimal place. Estimation options for the three programs may be different (e.g., the algorithm used to obtain estimates, number and spacing of quadrature points, rules for judging convergence of the algorithm, and so on) and may be responsible for such differences. Drastically larger discrepancies, should the user run estimation using more than one program, may be more indicative of an identification problem or poor starting values for estimation. Default approaches for estimating standard errors also vary across programs, and it is important for the user to choose an approach that is computationally feasible, but also reasonably accurate. Although it is outside the scope of this paper to make a particular recommendation, standard error estimation is the topic of much recent research (Tian et al., 2013; Paek and Cai, 2014; Pritikin, 2017; Chalmers, 2018).

### MNRM-Based Scoring

Once an appropriate model is estimated, scoring of individual participants may proceed. Such scores may be used to make decisions about individual participants or used in subsequent analyses (Curran et al., 2018). In what follows, we explain such scoring procedures in a heuristic way and refrain from the underlying mechanics (e.g., Thissen et al., 2001). However, estimated item parameters for category response functions in Equation (8) play an intricate role in determining an individual's score estimate on the underlying latent traits. Different values of item parameters would determine different shapes of category response functions (Figure 2). Responses to multiple items with a variety of category response function shapes then allows triangulation on the participant's location within the latent space. The below scoring procedures are illustrated with flexMIRT® in Supplementary Materials.

#### Response-Pattern-Based EAP Scores

The full response pattern can be used to obtain *Expected a Posteriori* (EAP) scores (Bock and Mislevy, 1982). In practice, maximum likelihood (ML) or *Maximum a Posteriori* (MAP) are also used, yet EAP scores are arguably easy for a computer to calculate from a multidimensional model and have good properties in terms of precision and recovery of scores. EAP scores are produced using a Bayesian approach that entails finding the mean of a posterior distribution for each participant. Of most importance to the current paper, we note that the posterior for EAP scores depends on the full response pattern. This means that two (or more) individuals may share the same sum scores on substantive and/or response style traits, but may have *different* EAP scores. Sum scores and pattern-based EAPs will diverge to the extent that item slopes and intercepts vary across items as some items may be better at differentiating among individuals at different levels of the latent trait(s). Table 2 provides several examples of this phenomena, including participants who share the same sum scores but different EAPs.

#### Sum-Score-Based EAP Scores

In contrast to EAP scores based on the full response pattern, sum-score-based EAP scores provide estimates of a posterior mean that only requires knowledge of an individuals' sum score. This means that individuals with the same sum scores will have the same estimated EAP scores under this approach. For instance, note in Table 2 how participants with the same sum score will also have the same sum-score-based EAP estimate. In addition, this approach facilitates pre-computation of sum score to EAP translation tables that may be used to provide an EAP score without the use of scoring software, which can be convenient in some applied settings. Sum-score-based EAPs provide estimates that can preserve some, but not all features of the non-linear relationship between the latent trait and the item responses. As details for sum score to EAP translation for response style models have not yet been previously presented, we provide additional details on this procedure in the Appendix at the end of this manuscript.

#### Summary of Scoring Results

**Figure 4:**
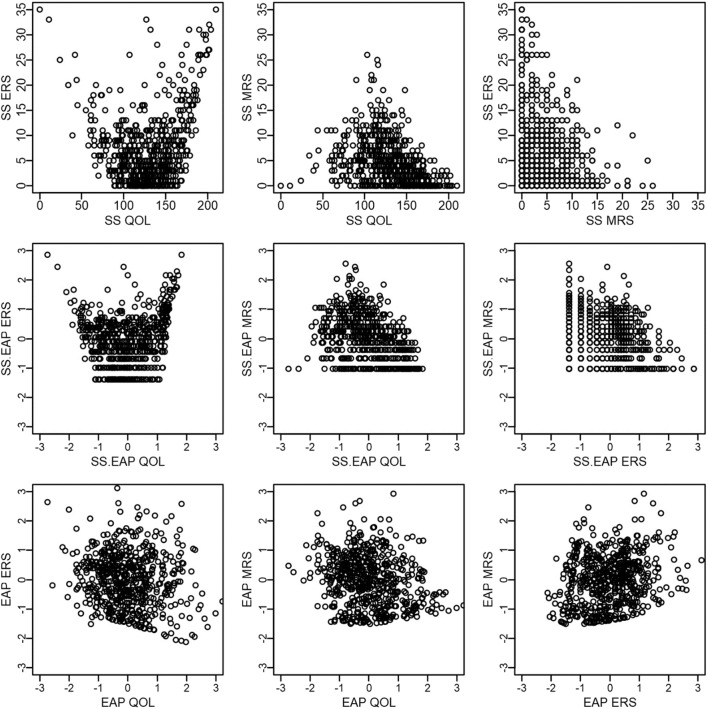
Scatter plots of QOL, ERS, and MRS scores within each scoring method.

Figure 4 presents scatterplots comparing scores within each scoring method, with the top row corresponding to sum scores, middle row to sum-score-based EAPs, and the bottom row to response-pattern-based EAPs. A few patterns are worth mentioning. First, QOL and ERS have a distinct U-shaped non-linear relationship for both sum scores and sum-score-based EAPs, such that those low or high on QOL tend to have high ERS scores. Such a pattern would be expected if it were difficult for such methods to disentangle ERS from the construct of interest. Another intuitive pattern arises for QOL and MRS—such that those who have intermediate QOL scores tend to have high MRS scores. Finally, a negative relationship is observed between ERS and MRS. Indeed, it is not possible for participants to use the endpoint categories at the same time as the middle categories. However, whether this negative relationship between ERS and MRS is due to an actual negative relationship between the two underlying constructs or is an artifact of the sum score procedure being confounded with substantive content is not immediately apparent until one examines response-pattern-based EAPs. This general pattern of results changes for response-pattern-based EAPs. That is, the scatterplots on the bottom row appear to depict clouds of points where a systematic pattern is more difficult to detect. This latter result is also consistent with estimated factor correlations from the fitted model that are somewhat small: −0.15 for QOL and ERS, −0.17 for QOL and MRS, and 0.18 for ERS and MRS.

**Figure 5:**
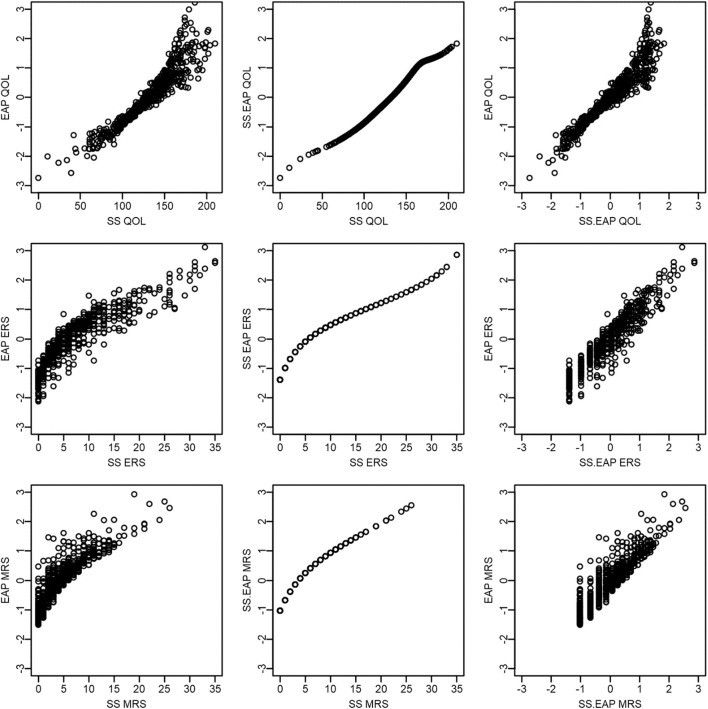
Scatter plots of QOL, ERS, and MRS scores across scoring methods.

Figure 5 compares across scoring methods, but within each latent dimension. The first row corresponds to QOL, the second to ERS, and the final to MRS. In general, there is a very strong positive relationship between the different scoring methods for the same dimension. Sum score and sum-score-based EAPs have a deterministic and non-linear relationship (the middle plot on each row; 0.95 < *rs* < 0.99). EAPs correlate strongly with both sum scores and sum-score-based EAPs, but have a far from perfect relationship. For instance, Pearson correlations between EAPs and sum scores are 0.94 for QOL, 0.89 for ERS, and 0.88 for MRS and between EAPs and sum-score-based EAPs are 0.95 for QOL, 0.94 for ERS, and 0.91 for MRS.

**Figure 6:**
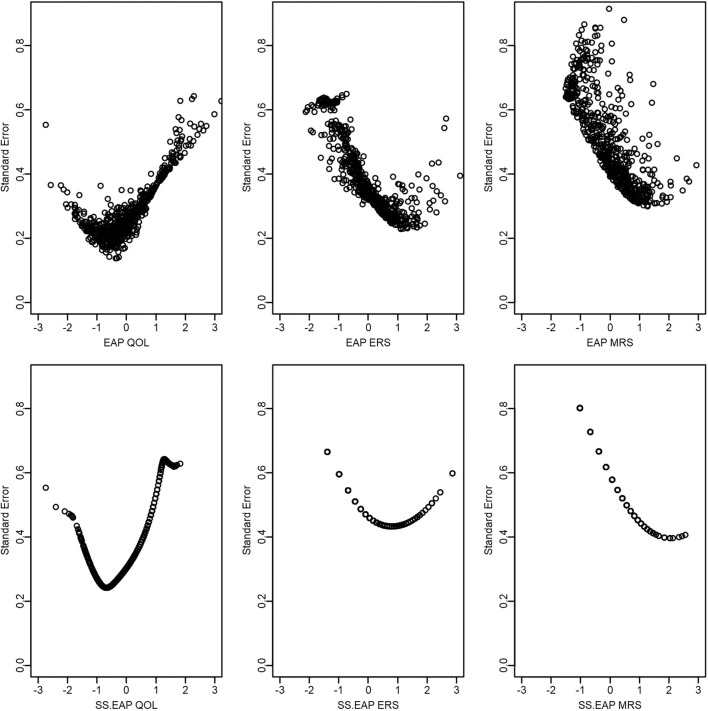
Scatter plots of QOL, ERS, and MRS scores and standard errors.

Finally, as an anonymous reviewer pointed out, both EAP and sum-score-based EAP scoring also provide standard errors for each score estimate (Figure 6). Such standard errors provide some potentially useful information regarding the precision of the score estimates, and sum scores do not readily provide this same information. For example, we see that standard errors for ERS and MRS tend to be larger than that for QOL, possibly indicating that it is more difficult to obtain accurate score estimates for response styles. In addition, standard errors for pattern EAP scores for ERS and MRS tend to be smaller than those based on sum scores. Standard errors also tend to be lowest at higher levels of ERS or MRS (e.g., between 1 and 2), suggesting that we have better score estimates for those who tend to be somewhat high on those constructs (relative to the mean the sample). In contrast, QOL appears to be most accurately measured for those who are slightly low on QOL (e.g., close to −1).

### Sum Score Equivalence: Separate Unidimensional Partial Credit Models

At the outset of our paper, we noted that it may be difficult to tell whether sum scores explicitly correspond to a particular measurement model. As obvious from the previous section, sum scores do not perfectly correspond to the modeling procedure outlined by Falk and Cai (2016), but provide a useful heuristic from which to scaffold to use of the MNRM. Since sum scores are sufficient statistics for estimating a unidimensional partial credit model (PCM; Masters, 1982), it is tempting to conclude that sum scores are equivalent to the procedure by Bolt and Newton (2011) in which slopes are equal across items or a variant that further constraints intercepts equal across items (a multidimensional rating scale model; Andrich, 1978)^3^. Aside from model fit deteriorating in some cases, neither of these approaches results in pattern-based EAP scores that are perfectly (albeit non-linearly) related to sum scores. The MNRM-based models that are most similar to sum scores are separate unidimensional models. Specifically, a unidimensional PCM using the scoring function for just QOL and constraining all item slopes to be equal would result in EAP scores that have a perfect Spearman rank correlation with sum scores for QOL. In addition, a unidimensional PCM with an ERS scoring function and equal slopes equal across items results in EAP scores with a perfect (non-linear) relationship with ERS sum scores. There does not appear to be a model that allows for simultaneous testing of sum-score-based approaches, making it impossible to evaluate use of sum scores based on grounds of model fit.

## Discussion and Conclusion

We have provided examples on the use and interpretation of the MNRM for measuring response styles, along with code for estimating the model and producing scores. We have also provided details on how sum-score-based EAP scores can be obtained from the MNRM. These examples were presented against the backdrop of comparing a popular sum score approach to two scoring methods based on the MNRM: response-pattern-based EAP and sum-score-based EAP.

Although the empirical example contained data from 35 items that are intended to measure a single underlying construct, EAP scores from the MNRM appeared to allow separation of style and content. In contrast, both sum scores and sum-score-based EAPs appeared to show a confounding of style and content. Estimates of the correlation among latent traits indicated that ERS and MRS were weakly related to each other and to QOL. These illustrations highlight that it is *insufficient* to merely examine correlations among sum scores for response styles and substantive constructs. Visual aids may help in identifying how style is confounded with content.

This result seemingly contrasts recommendations that response styles need to be measured from heterogeneous items (Greenleaf, 1992; De Beuckelaer et al., 2010). De Beuckelaer et al. (2010) refer to approaches that measure response styles using items from the substantive constructs of interest as “*ad-hoc*,” and are skeptical when style is measured using items with modest inter-item correlations (e.g., 0.20–0.27). Yet, we also do not dispute that a set of heterogeneous items may be best for measuring response styles and seek to reconcile this discrepancy. The MNRM may have a *relative* advantage over sum score approaches in separating style and content, and it is still advisable to have some items that are heterogeneous. In addition, the ability to have loadings and intercepts that vary across items may represent a distinct kind of heterogeneity. For instance, provided that items vary substantially in how easy they are to endorse and are also more or less strongly related to the substantive trait, a model-based approach such as the MNRM may be able to disentangle style from content from a set of items that are relatively homogenous in terms of content coverage.

Caution is warranted to not over-generalize the above results as the example was intended for didactic purposes in illustrating the MNRM. Assessing fit for such models is challenging, and we suspect that a better model may require more than one substantive dimension, and some previous illustrations have focused on bi-factor models for QOL (e.g., Gibbons et al., 2007). Thus, one might reasonably argue that the items used in this example do have some content heterogeneity. In addition, a full bi-factor nominal model reveals that lower categories for several QOL items may be indistinguishable or not perfectly ordinal. However, we note that the best fitting response style model studied here (QOL, ERS, and MRS) fits better according to AIC and BIC than a bi-factor model using the GPC or unconstrained nominal model. Furthermore, the addition of ERS and MRS to a bi-factor GPC model yields a similar pattern of EAP scores as in Figure 5^4^. Thus, one logical alternative modeling choice (bi-factor model with ERS and MRS) would have yielded similar conclusions. Still, it is possible that other measurement instruments, especially those with fewer items and response options, may not allow such a clean separation of style and content.

Conclusions regarding which approach is best must also be tied to evidence for validity, and we caution the reader that model fit is only one possible aspect. We present the MNRM with a concrete example that compares it to sum scores and provide examples and information to allow applied researchers to more widely use the model in practice for such validity investigations. We withhold arguing that the current approach is the best for modeling response styles, as there have recently emerged a number of alternatives (e.g., Plieninger and Meiser, 2014; Böckenholt and Meiser, 2017). However, the MNRM and the majority of similar latent trait models are most appropriate when there are multi-item measures of constructs, and typically assume what is known as a reflective measurement model (Bollen and Lennox, 1991). That is, it is reasonable to assume that underlying substantive constructs (and response styles) cause people to respond to items in a certain way. If either of these conditions does not hold, it may still be possible to use a latent model for extracting information about response styles, often from a separate set of items, and then make corrections to the [possibly sum score-based] responses for the substantive constructs of interest (Greenleaf, 1992; De Jong et al., 2008; van Rosmalen et al., 2010). In addition, more alternatives include the use of a separate dedicated set of items called anchoring vignettes (Bolt et al., 2014; Baird et al., 2017) or hybrid approaches where items that do not belong to the construct of interest are used to form sum score indicators for use in covariance structure analysis (Weijters et al., 2008). However, we note that researchers have done little to make comparisons among the plethora of extant response style models. A few examples provide some evidence for modeling approaches similar to what we use here (e.g., Deng et al., 2018; Schneider, 2018), but it is too early to draw definitive conclusions. The question of how to best disentangle response style from the construct(s) of interest thus remains an important issue, and we hope that the current manuscript will facilitate future comparisons with such alternatives.

## Appendix: Sum Score to EAP Translation

Here we provide details on sum-score to EAP translation for response styles and the Multidimensional Nominal Response Model (MNRM). We currently consider the case where all items load on the style dimensions. We follow similar notational conventions as that found in the main text associated with this paper.

For EAP scores (Bock and Mislevy, 1982), the mean of the posterior distribution is given by the following:

(10) $$\begin{array}{l} EAP\left(\text{x}\right)=\;\frac{\int \text{L}\left(\text{y}|\text{x}\right)\;\phi \;\left(\text{x}\right)\text{x}\text{d}\text{x}}{\int \text{L}\left(\text{y}|\text{x}\right)\;\phi \;\left(\text{x}\right)\text{d}\text{x}} \end{array}$$

where *L*(**y**|**x**) is the likelihood of the response pattern **y** given scores on the latent traits **x**, and ϕ(**x**) is a prior distribution—usually the multivariate normal density function based on the mean and variance-covariance of the latent traits. The integrals can be approximated using quadrature or Monte Carlo integration. Alternatively, with high-dimensional models, the mean of a large number of imputations from the posterior can be taken as the EAP estimate.

For sum-score-based EAPs, here we follow similar notational conventions as Cai (2015), though we do not consider item clusters nor seek to reduce the dimensions of integration as was done in this previous paper. For simplification, we also omit participant subscripts, *i*. Let **x** = [η **ξ**] indicate that the latent traits are partitioned into a factor of interest, η, and nuisance factors, **ξ**. In other words, η = *x*_*d*_, corresponds to some dimension *d* that is of interest for scoring purposes.

Explicit inclusion of custom item weights accomplishes the following two steps when conducting sum score to EAP scoring. First, weights, **s**_jd_, for item *j* on dimension *d* are used for recoding item responses. Buckley (2009) presents an expression for this recoding for response styles and that we modify for our purposes: $u_{jd}\left(y_{j}\right)=\overset{K_{j}-1}{\underset{k=0}{\sum }}s_{j,kd}1\left(\;{\text{y}}_{\text{j}}\;=\text{k}\right)$, where **1(***y*_*j*_ = *k***)** is an indicator function that equals one when *y*_*j*_ = *k* and zero otherwise. Such recoded variables are used to form sum scores for dimension *d*: $v_{d}=\;\overset{n}{\underset{j=1}{\sum }}u_{jd}\left(y_{j}\right)$. Second, we may re-define category response functions for dimension *d*,

(11) $$\begin{array}{l} T_{j}^{d}\left(m|\eta ,\xi \right)=\overset{K_{j}-1}{\underset{k=0}{\sum }}{1\left({\text{u}}_{\text{jd}}\left(\text{k}\right)=\text{ m}\right)T}_{j}\left(k|\eta ,\xi \right) \end{array}$$

where *m* ∈ **s**_*jd*_, and *T*_*j*_ (*k*|η, **ξ**) is short-hand notation for the category response function for item *j* (see Equation 8 in the main text). In other words, *m* is some possible recoding of the *K*_*j*_ categories for item *j*. For the examples in this paper, for a single item a participant can obtain an ERS score of “0” by selecting a category other than the endpoints, $T_{j}^{ERS}\left(0|\eta ,\xi \right)=T_{j}\left(1|\eta ,\xi \right)+T_{j}\left(2|\eta ,\xi \right)+T_{j}\left(3|\eta ,\xi \right)+T_{j}\left(4|\eta ,\xi \right)+T_{j}\left(5|\eta ,\xi \right)\;$, and a score of “1” by selecting an endpoint category, $T_{j}^{ERS}\left(1|\eta ,\xi \right)=T_{j}\left(0|\eta ,\xi \right)+T_{j}\left(6|\eta ,\xi \right)$. Note that flexMIRT® (Cai, 2017) does not automatically do such coding unless item weights are specified, even if the MNRM is used. Otherwise, item categories are treated as ordinal. As a result, custom item weights may only make sense for dimension *d*, multiple scoring runs may be needed if dimensions have different scoring functions, and scores for other dimensions may be ignored as necessary. In such a case, the model does not change—only the code used to extract sum scores converted to scale scores.

We then define the likelihood for **x** = [η **ξ**] for response pattern **y** and relevant for scoring dimension *d* as the following:

(12) $$\begin{array}{l} L^{d}\left(\text{y}|\eta ,\xi \right)=\overset{n}{\underset{j=1}{\prod }}T_{j}^{d}\left(u_{jd}\left(y_{j}\right)|\eta ,\xi \right) \end{array}$$

The sum-score-based likelihood for dimension *d* is then:

(13) $$\begin{array}{l} L^{d}\left(v_{d}|\eta ,\xi \right)=\;\underset{v_{d}=||\text{y}|{|}^{d}}{\sum }L^{d}\left(\text{y}|\eta ,\xi \right) \end{array}$$

where $||\text{y}|{|}^{d}=\overset{n}{\underset{j=1}{\sum }}u_{jd}\left(y_{j}\right)$ is short-hand notation for the sum score for dimension *d*, based on response pattern **y**. The sum in (13) is therefore over all response patterns that would yield sum score *v*_*d*_ on dimension *d*. Assuming a prior distribution, *ϕ* (*η*, **ξ**), the normalized joint posterior of **x** = [*η* **ξ**] for sum score *v*_*d*_ is:

(14) $$\begin{array}{l} p\left(\eta ,\xi |v_{d}\right)=\frac{L^{d}\left(v_{d}|\eta ,\xi \right)\phi \left(\eta ,\xi \right)}{p\left(v_{d}\right)} \end{array}$$

where *p*(*v*_*d*_) is the marginal probability of sum score *v*_*d*_:

(15) $$\begin{array}{l} p\left(v_{d}\right)=\iint L^{d}\left(v_{d}|\eta ,\xi \right)\phi \left(\eta ,\xi \right)d\xi d\eta . \end{array}$$

Since we are interested in the sum-score-based EAP score for only *η*, we may integrate the posterior over **ξ** to obtain a marginal posterior for *η*,

(16) $$\begin{array}{l} p\left(\eta |v_{d}\right)=\frac{1}{p\left(v_{d}\right)}\int L^{d}\left(v_{d}|\eta ,\xi \right)\phi \left(\eta ,\xi \right)d\xi \end{array}$$

and then further compute the expected value to obtain the EAP score for η,

(17) $$\begin{array}{l} E\left(\eta |v_{d}\right)=\frac{1}{p\left(v_{d}\right)}\int \eta \left[\int L^{d}\left(v_{d}|\eta ,\xi \right)\phi \left(\eta ,\xi \right)d\xi \right]d\eta \end{array}$$

Finally, the variance of this estimate, which can be used to form a standard error, can also be obtained:

(18) $$\begin{array}{l} V\left(\eta |v_{d}\right)=\;\frac{1}{p\left(v_{d}\right)}\int \eta^{2}\left[\int L^{d}\left(v_{d}|\eta ,\xi \right)\phi \left(\eta ,\xi \right)d\xi \right]d\eta \\ -E^{2}\left(\eta |v_{d}\right). \end{array}$$

In this paper, all integrals were approximated using rectangular quadrature with 49 equally spaced nodes from −6 to 6 along *each* latent dimension, with normalized quadrature weights, *W*(**x**), for **x** = [η ξ], taking the place of the multivariate normal density.

If items have more than two categories, a polytomous extension of the Lord-Wingersky algorithm (e.g., Cai, 2015) may be used to obtain the sum-score-based likelihoods, $L^{d}\left(v_{d}|\eta ,\xi \right)$. Consider the case of the model in the main text (see Figure 1) that included only QOL and ERS dimensions. Sum-score-based EAP estimates for ERS based on just the first three items may take the following procedure. Item parameter estimates for these items appear in Table 5. In addition, Table 6 lists quadrature nodes for nine different combinations of ERS (η) and QOL (ξ) along with normalized quadrature weights with a correlation of −0.18 among constructs. In addition, values for the category response functions for the MNRM based on the ERS dimension, $T_{j}^{ERS}\left(m|\eta ,\xi \right)$ for each item *j* and category *m* appear along this quadrature grid.

**Table 5:** Parameters for three items measuring QOL and ERS.

	**a^QOL^**	**a^ERS^**	**c_1_**	**c_2_**	**c_3_**	**c_4_**	**c_5_**	**c_6_**	**c_7_**
Item 1	0.80	1.68	0.00	1.84	2.39	3.57	3.94	3.64	2.49
Item 2	0.46	1.03	0.00	1.16	1.17	2.56	2.89	2.96	2.72
Item 3	0.49	1.49	0.00	1.50	1.97	2.30	2.95	2.65	1.61

**Table 6:** Ordinates of recoded category response functions and quadrature weights evaluated over the 3 × 3 direct product rectangular quadrature points for the three items.

η	**−2**	**−2**	**−2**	**0**	**0**	**0**	**2**	**2**	**2**
ξ	**−1**	**0**	**1**	**−1**	**0**	**1**	**−1**	**0**	**1**
*W*(**x**)	0.019	0.046	0.040	0.215	0.361	0.215	0.040	0.046	0.019
$T_{1}^{ERS}\left(0|\eta ,\xi \right)$	0.997	0.997	0.987	0.910	0.916	0.722	0.259	0.274	0.082
$T_{2}^{ERS}\left(0|\eta ,\xi \right)$	0.978	0.965	0.924	0.853	0.779	0.606	0.426	0.310	0.164
$T_{3}^{ERS}\left(0|\eta ,\xi \right)$	0.994	0.994	0.987	0.901	0.901	0.792	0.318	0.317	0.163
$T_{1}^{ERS}\left(1|\eta ,\xi \right)$	0.003	0.003	0.013	0.090	0.084	0.278	0.741	0.726	0.918
$T_{2}^{ERS}\left(1|\eta ,\xi \right)$	0.022	0.035	0.076	0.147	0.221	0.394	0.574	0.690	0.836
$T_{3}^{ERS}\left(1|\eta ,\xi \right)$	0.006	0.006	0.013	0.099	0.099	0.208	0.682	0.683	0.837

*η is for ERS (extreme response style), and ξ is for QOL (quality of life)*.

Then, as can be seen in Table 7, the algorithm begins with the initialization of sum-score-based likelihood with the first item,

$$\begin{array}{r} L_{1}^{ERS}\left(0|\eta ,\xi \right)=T_{1}\left(1|\eta ,\xi \right)+T_{1}\left(2|\eta ,\xi \right)\\ +T_{1}\left(3|\eta ,\xi \right)+T_{1}\left(4|\eta ,\xi \right)+T_{1}\left(5|\eta ,\xi \right)=T_{1}^{ERS}\left(0|\eta ,\xi \right)\\ L_{1}^{ERS}\left(1|\eta ,\xi \right)=T_{1}\left(0|\eta ,\xi \right)+T_{1}\left(6|\eta ,\xi \right)=T_{1}^{ERS}\left(1|\eta ,\xi \right), \end{array}$$

continues with additional recursions for the second item,

$$\begin{array}{r} L_{2}^{ERS}\left(0|\eta ,\xi \right)=L_{1}^{ERS}\left(0|\eta ,\xi \right)T_{2}^{ERS}\left(0|\eta ,\xi \right)\\ L_{2}^{ERS}\left(1|\eta ,\xi \right)=L_{1}^{ERS}\left(0|\eta ,\xi \right)T_{2}^{ERS}\left(1|\eta ,\xi \right)\\ +L_{1}^{ERS}\left(1|\eta ,\xi \right)T_{2}^{ERS}\left(0|\eta ,\xi \right)\\ L_{2}^{ERS}\left(2|\eta ,\xi \right)=L_{1}^{ERS}\left(1|\eta ,\xi \right)T_{2}^{ERS}\left(1|\eta ,\xi \right), \end{array}$$

and for the third item in the final step in Table 8. Subscripts are used to denote the sum score likelihood after adding each item, yet those from the last step, $L_{3}^{ERS}\left(\cdot |\eta ,\;\xi \right)$, are analogous to Equation (13) at each quadrature node. The “3” subscript is omitted at the top of Table 9, where the sum-score-based likelihoods are multiplied by quadrature weights. Approximation of the integral in Equation (16) requires summing across ξ for each unique node of η, resulting in the lower part of Table 5. These values may then be further summed to compute an approximation of $\left(v_{d}\right)\approx \underset{\eta }{\sum }\underset{\xi }{\sum }L^{ERS}\left(\nu_{d}|\eta ,\xi \right)W\left(\text{x}\right)$, or a weighted sum across nodes for η can be done to approximate integrals across η in Equations (17) and (18). For example, in Equation (17), $\int \eta \left[\int L^{d}\left(v_{d}|\eta ,\xi \right)\phi \left(\eta ,\xi \right)d\xi \right]d\eta \approx \underset{\eta }{\sum }\eta \left[\underset{\xi }{\sum }L^{ERS}\left(\nu_{d}|\eta ,\xi \right)W\left(\text{x}\right)\right]$. Final example EAP scores and variance estimates appear in Table 9.

**Table 7:** Accumulating sum score likelihoods.

**Quadrature grid for (η,** **ξ)**										
**INITIALIZATION OF SUM SCORE LIKELIHOODS WITH ITEM 1**										
η		−2	−2	−2	0	0	0	2	2	2
ξ		−1	0	1	−1	0	1	−1	0	1
$L_{1}^{ERS}\left(0|\eta ,\;\xi \right)=$	$T_{1}^{ERS}\left(0|\eta ,\xi \right)$	0.997	0.997	0.987	0.910	0.916	0.722	0.259	0.274	0.082
$\;L_{1}^{ERS}\left(1|\eta ,\;\xi \right)=$	$T_{1}^{ERS}\left(1|\eta ,\xi \right)$	0.003	0.003	0.013	0.090	0.084	0.278	0.741	0.726	0.918
**ADDING ITEM 2 TO EXISTING SUM SCORE LIKELIHOODS**										
$L_{2}^{ERS}\left(0|\eta ,\;\xi \right)=$	$L_{1}^{ERS}\left(0|\eta ,\xi \right)T_{2}^{ERS}\left(0|\eta ,\xi \right)$	0.975	0.962	0.911	0.776	0.713	0.438	0.110	0.085	0.014
$L_{2}^{ERS}\left(1|\eta ,\;\xi \right)=$	$L_{1}^{ERS}\left(0|\eta ,\xi \right)T_{2}^{ERS}\left(1|\eta ,\xi \right)+L_{1}^{ERS}\left(1|\eta ,\xi \right)T_{2}^{ERS}\left(0|\eta ,\xi \right)$	0.025	0.038	0.088	0.211	0.268	0.453	0.464	0.414	0.220
$L_{2}^{ERS}\left(2|\eta ,\;\xi \right)=$	$L_{1}^{ERS}\left(1|\eta ,\xi \right)T_{2}^{ERS}\left(1|\eta ,\xi \right)$	0.000	0.000	0.001	0.013	0.019	0.110	0.426	0.501	0.767
**ADDING ITEM 3 TO EXISTING SUM SCORE LIKELIHOODS**										
$L_{3}^{ERS}\left(0|\eta ,\;\xi \right)=$	$L_{2}^{ERS}\left(0|\eta ,\xi \right)T_{3}^{ERS}\left(0|\eta ,\xi \right)$	0.970	0.957	0.899	0.700	0.643	0.347	0.035	0.027	0.002
$L_{3}^{ERS}\left(1|\eta ,\;\xi \right)=$	$L_{2}^{ERS}\left(0|\eta ,\xi \right)T_{3}^{ERS}\left(1|\eta ,\xi \right)+L_{2}^{ERS}\left(1|\eta ,\xi \right)T_{3}^{ERS}\left(0|\eta ,\xi \right)$	0.030	0.043	0.099	0.266	0.312	0.450	0.223	0.189	0.047
$L_{3}^{ERS}\left(2|\eta ,\;\xi \right)=$	$L_{2}^{ERS}\left(1|\eta ,\xi \right)T_{3}^{ERS}\left(1|\eta ,\xi \right)+L_{2}^{ERS}\left(2|\eta ,\xi \right)T_{3}^{ERS}\left(0|\eta ,\xi \right)$	0.000	0.000	0.002	0.033	0.043	0.181	0.452	0.442	0.309
$L_{3}^{ERS}\left(3|\eta ,\;\xi \right)=$	$L_{2}^{ERS}\left(2|\eta ,\xi \right)T_{3}^{ERS}\left(1|\eta ,\xi \right)$	0.000	0.000	0.000	0.001	0.002	0.023	0.290	0.342	0.642

**Table 8:** Adding weights and integrating over the nuisance dimension.

	**Quadrature grid for (η, ξ)**								
η	**−2**	**−2**	**−2**	**0**	**0**	**0**	**2**	**2**	**2**
ξ	**−1**	**0**	**1**	**−1**	**0**	**1**	**−1**	**0**	**1**
**MULTIPLY SUM SCORE LIKELIHOODS BY WEIGHTS *W*(**x**)**									
*L*^*ERS*^(0|η, ξ)*W*(**x**)	0.018	0.044	0.036	0.151	0.232	0.075	0.001	0.001	0.000
*L*^*ERS*^(1|η, ξ)*W*(**x**)	0.001	0.002	0.004	0.057	0.113	0.097	0.009	0.009	0.001
*L*^*ERS*^(2|η, ξ)*W*(**x**)	0.000	0.000	0.000	0.007	0.016	0.039	0.018	0.020	0.006
*L*^*ERS*^(3|η, ξ)*W*(**x**)	0.000	0.000	0.000	0.000	0.001	0.005	0.011	0.016	0.012
	η									
	−2			0			2		
**SUMMING OVER** ξ									
$\underset{\xi }{\sum }L^{ERS}\left(0|\eta ,\xi \right)W\left(\text{x}\right)$	0.098			0.457			0.003		
$\underset{\xi }{\sum }L^{ERS}\left(1|\eta ,\xi \right)W\left(\text{x}\right)$	0.006			0.267			0.018		
$\underset{\xi }{\sum }L^{ERS}\left(2|\eta ,\xi \right)W\left(\text{x}\right)$	0.000			0.062			0.044		
$\underset{\xi }{\sum }L^{ERS}\left(3|\eta ,\xi \right)W\left(\text{x}\right)$	0.000			0.006			0.039		

**Table 9:** Final score and variance estimates.

**Sum Scores**	**Posterior Summaries**		
	**p**(ν_ERS_)	**E**(η|ν_ERS_)	**V**(η|v_ERS_)
ν_*ERS*_ = 0	0.558	−0.340	0.603
ν_*ERS*_ = 1	0.292	0.082	0.333
ν_*ERS*_ = 2	0.106	0.829	0.979
ν_*ERS*_ = 3	0.045	1.740	0.452

## Data Availability Statement

All datasets generated for this study are included in the article/supplementary material.

## Author Contributions

CF and UJ drafted the paper idea, decided on an empirical example and plan for the paper, and split work on initial analyses. CF performed the initial draft of most aspects of the paper, Supplementary Materials, and graphics. UJ completed tables for sum score to EAP translation, and also edited the manuscript and Supplementary Materials.

### Conflict of Interest

UJ is employed at Riverside Insights. Her prior affiliation during the majority of work on the paper was at Michigan State University. The remaining authors declare that the research was conducted in the absence of any commercial or financial relationships that could be construed as a potential conflict of interest.

## Footnotes

^1^The logic underlying this representation can be found in other resources (e.g., Thissen et al., 2010) and provides additional flexibility for the MNRM.

^2^We use a tilde here to avoid notational confusion with “a” from the Thissen and Cai (2016) parameterization, though it is common to use “a” with the original nominal categories model.

^3^Constraints to estimate these models appear in our flexMIRT® control files and are commented out.

^4^Results of these models are available upon request.
