# Supplementary material for: Estimation of Response Styles Using the Multidimensional Nominal Response Model: A Tutorial and Comparison With Sum Scores
Source: Front Psychol. 2020 Feb 6;11:72. doi: 10.3389/fpsyg.2020.00072 (PMC7017717; doi:10.3389/fpsyg.2020.00072)
Supplement: Supplementary file 1 [file Data_Sheet_1.zip › AnnotatedControlFiles.pdf]

Supplementary Materials for *Estimation of response styles using the multidimensional nominal response model: A tutorial and comparison with sum scores*

Carl F. Falk
McGill University

Unhee Ju
Michigan State University

Supplementary Materials for *Estimation of response styles using the multidimensional nominal response model: A tutorial and comparison with sum scores*

In these supplementary materials, we provide in the following order: 1) A description of and annotated control files for estimation of the multidimensional nominal response model (MNRM) using flexMIRT® (Cai, 2017); 2) R code to estimate the MNRM with the *mirt* package in R (Chalmers, 2012); and 3) Control files to estimate the MNRM with *Mplus* (Muthén & Muthén, 2008).

1 flexMIRT

In this section, we focus mainly on code necessary for estimating a constrained MNRM with response styles and refer readers elsewhere for documentation pertaining to fitting basic item response models and other aspects of model syntax (Houts & Cai, 2016). Control files for all models used for calibration and scoring along with output files (with "-irt", "-prm", "-sco", "-ssc" extensions) also appear in separate files.

1.1 Model Specification and Estimation

First, consider "QOL_Calib_1dimERS.txt", which estimates the model in Figure 1 in the main text. Under the `<Options>` section, line 6 (`Mode = Calibration`) specifies that running this control file results in estimates of model parameters, and such parameters are saved with line 10 (`SavePRM = Yes`) to a file with a "-prm" suffix for possible later use in computing scores using more than one alternative approach. The data set (QOL.dat; line 19) should be in the same folder as the control file and contains 35 items in columns separated by a tab character (space or comma is also acceptable) and without column headers. We use short-hand to label items as y1 through y35 (`Varnames = y1-y35;` line 20). We also specify that each item has seven categories (`Ncats(y1-y35) = 7;` line 21) and that each item should be modeled with the nominal response model with seven response options (`Model(y1-y35) = Nominal(7);` line 22). Line 22 (`Dimensions = 2`) additionally specifies that the model has two latent dimensions. When maximizing the log-likelihood using the EM algorithm (Bock & Aitkin, 1981), the latent traits are often assumed multivariate normal, standardized with mean zero and variance one for identification of the latent scale. Provided it does not cause identification problems, the covariance among these latent traits can be estimated (e.g., line 41).

Finally, custom scoring functions must be specified such that one dimension represents QOL and the other ERS. The scoring functions of the MNRM are not directly estimated, but are a product of a $K \times (K-1)$ matrix, $\mathbf{T}_a$, and a $(K-1)$ vector, $\boldsymbol{\alpha}$, for each dimension. That is, $\mathbf{s}_d = \mathbf{T}_{a,d}\boldsymbol{\alpha}_d$ for each dimension, d. The logic underlying this representation can be found in other resources (e.g., Thissen, Cai, & Bock, 2010), but in short provides much flexibility in the types of item models that can be specified under the MNRM. Here, custom scoring functions are not too difficult to specify, however, and require two steps. First, line 42 (`Fix (y1-y35), ScoringFn`) specifies that the scoring function values for each item should be fixed and not estimated ($\boldsymbol{\alpha}_d$ is fixed to a vector of length 6 with 1 as its first element, and 0 elsewhere). Second, we can then proceed to specify custom scoring functions by explicitly providing custom $\mathbf{T}_a$ matrices for each latent dimension (lines 24 through 39). Since $\boldsymbol{\alpha}_d$ is fixed for each dimension, all that is required for custom scoring functions is to explicitly list the scoring function

values in the first column of each $\mathbf{T}_{a,d}$ matrix. The full "`Ta(y1-y35)`" input resembles a block-diagonal super-matrix:

$$\begin{bmatrix} \mathbf{T}_{a,1} & \mathbf{0} \\ \mathbf{0} & \mathbf{T}_{a,2} \end{bmatrix}$$

where $\mathbf{0}$ is a matrix of zeros of appropriate dimensions. In the input file, commas separate each row, and a space separates each column. For instance, note the values 0 through 6 appearing in the first column and first seven rows of this input; these values correspond to the custom scoring function for the substantive (QOL) dimension. Thus, the $\mathbf{T}_{a,1}$ matrix for the first latent dimension spans lines 25-31 and the first six columns of this input (a 7×6 matrix), and corresponds to the following:

$$\mathbf{T}_{a,1} = \begin{bmatrix} 0 & 0 & 0 & 0 & 0 & 0 \\ 1 & 0 & 0 & 0 & 0 & 0 \\ 2 & 0 & 0 & 0 & 0 & 0 \\ 3 & 0 & 0 & 0 & 0 & 0 \\ 4 & 0 & 0 & 0 & 0 & 0 \\ 5 & 0 & 0 & 0 & 0 & 0 \\ 6 & 0 & 0 & 0 & 0 & 0 \end{bmatrix}$$

Zeros appear in the latter five columns of this input. Since $\boldsymbol{\alpha}_d' = \begin{bmatrix} 1 & 0 & 0 & 0 & 0 & 0 \end{bmatrix}$ is fixed and only its first element has a non-zero value, only the first column of $\mathbf{T}_{a,1}$ and $\mathbf{T}_{a,2}$ are relevant for defining the category response functions. Only when other elements of $\boldsymbol{\alpha}_d$ are non-zero, and/or $\boldsymbol{\alpha}_d$ is estimated (e.g., as part of the full nominal model) will the other columns of $\mathbf{T}_{a,1}$ and $\mathbf{T}_{a,2}$ be useful to the researcher (e.g., see Thissen, Cai, & Bock, 2010). The next $\mathbf{T}_{a,2}$ matrix spans the next seven rows and six columns (from lines 32-38 and columns 7-12),

$$\mathbf{T}_{a,2} = \begin{bmatrix} 1 & 0 & 0 & 0 & 0 & 0 \\ 0 & 0 & 0 & 0 & 0 & 0 \\ 0 & 0 & 0 & 0 & 0 & 0 \\ 0 & 0 & 0 & 0 & 0 & 0 \\ 0 & 0 & 0 & 0 & 0 & 0 \\ 0 & 0 & 0 & 0 & 0 & 0 \\ 1 & 0 & 0 & 0 & 0 & 0 \end{bmatrix}$$

The first column of this matrix contains the values, $\begin{bmatrix} 1 & 0 & 0 & 0 & 0 & 0 & 1 \end{bmatrix}$, representing the scoring function values for ERS.

If additional dimensions (substantive or response style), the super-matrix provided as input can be expanded. For instance, suppose we add an MRS factor (Figure 3), with scoring function $\begin{bmatrix} 0 & 0 & 0 & 1 & 0 & 0 & 0 \end{bmatrix}$. This is accomplished in an analogous manner in the "QOL_Calib_1dimERSMRS.txt" control file. Specifically, lines 38-44 define the scoring functions for MRS. Note how there is only a single "1" in the first column for the

MRS dimension (column 13), but zeros elsewhere. If scoring functions need to be specified in a different way across items either for substantive reasons or a different number of categories per item, multiple statements defining $\mathbf{T}_a$ matrices (with use of different item labels) can be provided. For example, this input might start with "`Ta(y1-y12)`" for items one through twelve, and "`Ta(y13-y35)`" for the remaining items.

The output file with an "-irt" suffix is the main output file containing summary information regarding calibration results. Item slopes are the first reported item parameters. For example, in "QOL_Calib_1dimERS-irt.txt", Item 2 has values of $a_{QOL} = 0.46$ and $a_{ERS} = 1.03$ when only these two dimensions are modeled (Figure 2). To ensure that scoring functions were specified as desired, the output will also typically have a section labeled "Nominal Model Scoring Function Values (s) under Dimension d" where "d" is the number for a latent dimension. Users can check that the desired scoring function, $\mathbf{s}_{jd}$, matches the values of this output. In the current example, the values, $\begin{bmatrix} 0 & 1 & 2 & 3 & 4 & 5 & 6 \end{bmatrix}$, appear for each item for Dimension 1 (i.e., QOL), but the values $\begin{bmatrix} 1 & 0 & 0 & 0 & 0 & 0 & 1 \end{bmatrix}$ appear for each item for Dimension 2 (i.e., ERS). In addition, the "Latent Variable Variance-Covariance Matrix" shows that the estimated correlation between QOL and ERS is small and negative ($-.18$).

Estimated intercepts are not often directly interpretable without additional work. Specifically, intercepts are also estimated as a matrix and vector product, $\mathbf{c} = \mathbf{T}_c\boldsymbol{\gamma}$, where $\mathbf{T}_c$ is a $K \times (K-1)$ matrix of full column rank, and $\boldsymbol{\gamma}$ is a $(K-1)$ length vector of estimated parameters – the latter of which is what actually appears in flexMIRT® output. Additional details are given by others (Falk & Cai, 2016; Thissen & Cai, 2016). In practice, it may be sufficient to report $\boldsymbol{\gamma}$ and just explicitly state that these are the "gammas" reported in the flexMIRT® output file.

We have not told flexMIRT® which items load on which factors; by default, all items load on all factors unless the user makes explicit requests in the `<Constraints>` section. A typical strategy to accomplish this task often involves fixing slopes for items on all dimensions to zero, and then free particular slopes later in the same constraint section. Although we do not discuss this procedure at length here, extant examples appear in the flexMIRT® manual (e.g., Section 4.2; Houts & Cai, 2016, p. 65-68) and support files (e.g., Example 4-3; https://www.vpgcentral.com/software/irt-software/support/).

In addition, it is possible to constrain the slopes for some dimensions equal across items, analogous to the model used by Bolt and Newton (2011). For an example, see lines 46–47 of the "QOL_Calib_1dimERS.txt" file, which could be uncommented to constrain the slopes for dimension 1 (QOL) or 2 (ERS) equal across items, respectively. If a rating scale type model is desired, additional equality constraints could be placed on intercepts across items (see lines 50 through 55).

To obtain the limited information fit statistic, M_2, line 13 of the "QOL_Calib_1dimERS.txt" file should be uncommented. However, note that use of multicore processing may sometimes result in small numerical differences in runs to compute M_2. To additionally obtain a Tucker-Lewis index, line 14 should also be uncommented as this compares the fitted model to the null model. Note, however, that the computation of M_2 is slow for this example. Other limited information test statistics do additional collapsing of marginal tables when items are ordinal and are more computationally feasible (Cai &

Monroe, 2013; Cai & Hansen, 2013). However, it is unclear whether these alternative are appropriate for nominal items. Currently, we suspect the performance of M_2 may not be optimal with many items and many categories per item. The output files also contain local dependence statistics (e.g., Chen & Thissen, 1997) for which there are extant examples for interpretation (e.g., Hansen et al., 2014).

1.2 Scoring

Once the item response model is estimated, EAP scoring or sum-score-based EAP scoring with unique category scores for each item and dimension can be conducted using flexMIRT®. Scoring can be performed immediately with the same control file that performs calibration by additionally setting flexMIRT® options to save scores and by providing an appropriate scoring method. For example, see "QOL_Calib_1dimERSMRS.txt" in which scores are requested (`SaveSCO = Yes;` line 9) and EAP is chosen as the scoring method (`Score = EAP;` line 8). These scores are saved to a file with an "-sco" suffix, with the columns of this file indicating the group ID, respondent ID, scores for each dimension, and the elements of the variance-covariance matrix for the scores (which could be used to form standard errors).

Scoring can also be performed post-calibration by reading in item parameter estimates from a previous calibration run. For example, see "QOL_SSEAP_QOL.txt" for an example in which sum-score-based EAPs are computed for the QOL dimension. Line 6 (`Mode = Scoring`) in the `<Options>` section indicates that the program will only perform scoring and will not estimate item parameters. This requires that item parameter estimates are read in from a "-prm" file (flexMIRT® generated or manually created) by setting the file name under the `<Options>` section on line 9 (`ReadPRMFile = "QOL_Calib_1dimERSMRS-prm.txt"`). Here, `Score = SSC` (line 7) indicates that sum-score-based EAP scores will be computed. Note that it is usually a good idea that much of the `<Groups>` section matches that used for calibration, though the data file for the scoring fun may be different and the full $\mathbf{T}_a$ matrix specification is usually not required as these matrices are saved in the "-prm" file. If variable names in the scoring control file do not match those initially used for calibration, flexMIRT® will not be able to match estimates in the parameter file ("-prm") with the items in the data file containing item responses. To double-check that the correct parameter estimates were used, the user may examine the "-ssc" file generated upon completion of scoring. In the case of sum-score-based EAPs, this file will also contain an appropriate translation table under the heading "Summed Score to Scale Score Conversion Table."

If not explicitly set in the control file, the initial category order (0 through $K_j - 1$) are used for computing scores for sum score to EAP translation. Thus, scoring functions are not automatically utilized to recode variables and specification of such recoding is not present in "QOL_SSEAP_QOL.txt" as the initial ordering already corresponds to the QOL dimension. Only the first scored dimension in both the "-sco" and score conversion table is therefore appropriate for interpretation as the QOL dimension. To obtain ERS and MRS scores, the files "QOL_SSEAP_ERS.txt" and "QOL_SSEAP_MRS.txt" provide examples where recoding is used for sum score to EAP translation. For example, line 19 of this first file contains `ItemWeights(y1-y35) = (1.0,0,0,0,0,0,1.0),` which specifies ERS recoding for all 35 items. Sum-score-based EAPs can then be extracted

from the second (ERS) and third (MRS) columns of EAP scores of the “QOL_SSEAP_ERS-sco.txt” and “QOL_SSEAP_MRS-sco.txt” files, respectively.

1.3 Numbered Control files

The following sections contain selected annotated control files including line numbers and additional comments that may be useful for understanding each control file and what each line of the control file does. Comments begin with "//" and continue until the end of a line.

Calibration: Quality of Life with Extreme Response Style

The following contains line numbers for "QOL_Calib_1dimERS.txt".

```
<Project>
Title = "QOL Data";
Description = "1 dimension + extreme response style";

<Options>
  Mode = Calibration;   // Estimate parameters. Other options include Scoring and Simulation.
  GOF = Extended;       // Goodness of fit statistics. Extended or Complete required for M2.
  Score = EAP;          // Provides scores using the EAP method. Other options include MAP, ML, SSC.
  SaveSCO = Yes;        // Save scores to a file with "-sco.txt" extension.
  SavePRM = Yes;        // Saves the parameter estimates in "-prm.txt" file. Useful for later reading back in to flexMIRT.
  Processors = 6;       // Will use 6 cores of a multiple core processor (most modern computers) to make estimation go faster.
  NewThreadModel = Yes; // Possibly faster multicore processing.
  //M2 = Full;          // Uncomment to compute M2. Full is based on the full bivariate margins (Maydeu-Olivares & Joe, 2005).
  //FitNullModel = Yes; // Uncomment to compute the null model used for TLI.

<Groups>

%Group1%                       // A name for the group. In this example we have only one group.
  File = "QOL.dat";            // The name of the data file that contains item responses.
  Varnames = y1-y35;           // Variable names for the data file. Short-hand is used to define v1 through v35.
  Ncats(y1-y35) = 7;           // Number of categories for variables y1-y35. Multiple Ncats statements are permitted.
  Model(y1-y35) = Nominal(7); // Item response model to use for variables y1-y35. Nominal model with 7 categories.
  Dimensions = 2;              // Number of latent dimensions. Here we have 2: QOL and ERS.
  Ta(y1-y35) = (               // Begins definition of Ta matrices for each dimension for variables y1-y35.
    0 0 0 0 0 0 0 0 0 0 0 0,  // k=0 for first dimension (QOL; first 6 columns).
    1 0 0 0 0 0 0 0 0 0 0 0,  // k=1 for first dimension (QOL; first 6 columns).
    2 0 0 0 0 0 0 0 0 0 0 0,  // k=2 for first dimension (QOL; first 6 columns).
    3 0 0 0 0 0 0 0 0 0 0 0,  // k=3 for first dimension (QOL; first 6 columns).
    4 0 0 0 0 0 0 0 0 0 0 0,  // k=4 for first dimension (QOL; first 6 columns).
    5 0 0 0 0 0 0 0 0 0 0 0,  // k=5 for first dimension (QOL; first 6 columns).
    6 0 0 0 0 0 0 0 0 0 0 0,  // k=6 for first dimension (QOL; first 6 columns).
    0 0 0 0 0 0 1 0 0 0 0 0,  // k=0 for second dimension (ERS; columns 7-12).
    0 0 0 0 0 0 0 0 0 0 0 0,  // k=1 for second dimension (ERS; columns 7-12).
    0 0 0 0 0 0 0 0 0 0 0 0,  // k=2 for second dimension (ERS; columns 7-12).
    0 0 0 0 0 0 0 0 0 0 0 0,  // k=3 for second dimension (ERS; columns 7-12).
    0 0 0 0 0 0 0 0 0 0 0 0,  // k=4 for second dimension (ERS; columns 7-12).
    0 0 0 0 0 0 0 0 0 0 0 0,  // k=5 for second dimension (ERS; columns 7-12).
    0 0 0 0 0 0 1 0 0 0 0 0   // k=6 for second dimension (ERS; columns 7-12).
);                             // End of Ta matrix definitions.

<Constraints>
  Fix (y1-y35),ScoringFn;    // Specifies that all scoring function values are fixed, not estimated.
  Free Cov(2,1);             // Frees covariance between dimensions 1 and 2.

```

```
// Uncomment the following two lines to fit a partial credit model analogous to that by Bolt and Newton (2011)
//  Equal Group1, (y1-y35), Slope (1); // Uncomment to set all QOL slopes equal
//  Equal Group1, (y1-y35), Slope (2); // Uncomment to set all ERS slopes equal

// To also constrain intercepts equal, uncomment the following lines
//  Equal Group1, (y1-y35), Intercept(1); // All intercept 1's equal
//  Equal Group1, (y1-y35), Intercept(2); // All intercept 2's equal
//  Equal Group1, (y1-y35), Intercept(3); // All intercept 3's equal
//  Equal Group1, (y1-y35), Intercept(4); // All intercept 4's equal
//  Equal Group1, (y1-y35), Intercept(5); // All intercept 5's equal
//  Equal Group1, (y1-y35), Intercept(6); // All intercept 6's equal
```

Calibration: Quality of Life with Extreme and Midpoint Response Style

The following contains line numbers for "QOL_Calib_1dimERSMRS.txt".

```
<Project>
Title = "QOL Data";
Description = "1 dimension + ERS and MRS";

<Options>
  Mode = Calibration;   // Estimate parameters. Other options include Scoring and Simulation.
  GOF = Extended;       // Goodness of fit statistics. Extended or Complete required for M2.
  Score = EAP;          // Provides scores using the EAP method. Other options include MAP, ML, SSC.
  SaveSCO = Yes;        // Save scores to a file with "-sco.txt" extension.
  SavePRM = Yes;        // Saves the parameter estimates in "-prm.txt" file. Useful for later reading back in to flexMIRT.
  Processors = 6;       // Will use 6 cores of a multiple core processor (most modern computers) to make estimation go faster.
  NewThreadModel = Yes; // Possibly faster multicore processing.
  //M2 = Full;          // Uncomment to compute M2. Full is based on the full bivariate margins (Maydeu-Olivares & Joe, 2005).
  //FitNullModel = Yes; // Uncomment to compute the null model used for TLI.

<Groups>

%Group1%
  File = "QOL.dat";           // The name of the data file that contains item responses.
  Varnames = y1-y35;          // Variable names for the data file. Short-hand is used to define y1 through y35.
  Ncats(y1-y35) = 7;          // Number of categories for variables y1-y35. Multiple Ncats statements are permitted.
  Model(y1-y35) = Nominal(7); // Item response model to use for variables y1-y35. Nominal model with 7 categories.
  Dimensions = 3;             // Number of latent dimensions. Here we have 3: QOL, ERS, MRS.
  Ta(y1-y35) = (              // Begins definition of Ta matrices for each dimension for variables y1-y35.
    0 0 0 0 0 0 0 0 0 0 0 0 0 0 0 0 0 0 0 0 0,  // k=0 for first dimension (QOL; first 6 columns).
    1 0 0 0 0 0 0 0 0 0 0 0 0 0 0 0 0 0 0 0 0,  // k=1 for first dimension (QOL; first 6 columns).
    2 0 0 0 0 0 0 0 0 0 0 0 0 0 0 0 0 0 0 0 0,  // k=2 for first dimension (QOL; first 6 columns).
    3 0 0 0 0 0 0 0 0 0 0 0 0 0 0 0 0 0 0 0 0,  // k=3 for first dimension (QOL; first 6 columns).
    4 0 0 0 0 0 0 0 0 0 0 0 0 0 0 0 0 0 0 0 0,  // k=4 for first dimension (QOL; first 6 columns).
    5 0 0 0 0 0 0 0 0 0 0 0 0 0 0 0 0 0 0 0 0,  // k=5 for first dimension (QOL; first 6 columns).
```

```
    6 0 0 0 0 0 0 0 0 0 0 0 0 0 0 0 0 0,  // k=6 for first dimension (QOL; first 6 columns).
    0 0 0 0 0 0 1 0 0 0 0 0 0 0 0 0 0 0,  // k=0 for second dimension (ERS; columns 7-12).
    0 0 0 0 0 0 0 0 0 0 0 0 0 0 0 0 0 0,  // k=1 for second dimension (ERS; columns 7-12).
    0 0 0 0 0 0 0 0 0 0 0 0 0 0 0 0 0 0,  // k=2 for second dimension (ERS; columns 7-12).
    0 0 0 0 0 0 0 0 0 0 0 0 0 0 0 0 0 0,  // k=3 for second dimension (ERS; columns 7-12).
    0 0 0 0 0 0 0 0 0 0 0 0 0 0 0 0 0 0,  // k=4 for second dimension (ERS; columns 7-12).
    0 0 0 0 0 0 0 0 0 0 0 0 0 0 0 0 0 0,  // k=5 for second dimension (ERS; columns 7-12).
    0 0 0 0 0 0 1 0 0 0 0 0 0 0 0 0 0 0,  // k=6 for second dimension (ERS; columns 7-12).
    0 0 0 0 0 0 0 0 0 0 0 0 0 0 0 0 0 0,  // k=0 for third dimension (MRS; columns 13-18).
    0 0 0 0 0 0 0 0 0 0 0 0 0 0 0 0 0 0,  // k=1 for third dimension (MRS; columns 13-18).
    0 0 0 0 0 0 0 0 0 0 0 0 0 0 0 0 0 0,  // k=2 for third dimension (MRS; columns 13-18).
    0 0 0 0 0 0 0 0 0 0 0 0 1 0 0 0 0 0,  // k=3 for third dimension (MRS; columns 13-18).
    0 0 0 0 0 0 0 0 0 0 0 0 0 0 0 0 0 0,  // k=4 for third dimension (MRS; columns 13-18).
    0 0 0 0 0 0 0 0 0 0 0 0 0 0 0 0 0 0,  // k=5 for third dimension (MRS; columns 13-18).
    0 0 0 0 0 0 0 0 0 0 0 0 0 0 0 0 0 0   // k=6 for third dimension (MRS; columns 13-18).
);                            // End of Ta matrix definitions.

<Constraints>
  Fix (y1-y35),ScoringFn;    // Specifies that all scoring function values are fixed, not estimated.
  Free Cov(1,2);             // Frees covariance between dimensions 1 and 2.
  Free Cov(1,3);             // Frees covariance between dimensions 1 and 3.
  Free Cov(2,3);             // Frees covariance between dimensions 2 and 3.

// Uncomment the following three lines to fit a partial credit model analogous to that by Bolt and Newton (2011)
//  Equal Group1, (y1-y35), Slope (1); // Uncomment to set all QOL slopes equal
//  Equal Group1, (y1-y35), Slope (2); // Uncomment to set all ERS slopes equal
//  Equal Group1, (y1-y35), Slope (3); // Uncomment to set all MRS slopes equal

// To also constrain intercepts equal, uncomment the following lines
//  Equal Group1, (y1-y35), Intercept(1); // All intercept 1's equal
//  Equal Group1, (y1-y35), Intercept(2); // All intercept 2's equal
//  Equal Group1, (y1-y35), Intercept(3); // All intercept 3's equal
//  Equal Group1, (y1-y35), Intercept(4); // All intercept 4's equal
//  Equal Group1, (y1-y35), Intercept(5); // All intercept 5's equal
//  Equal Group1, (y1-y35), Intercept(6); // All intercept 6's equal
```

Sum score to EAP translation: Quality of Life

The following contains line numbers for "QOL_SSEAP_QOL.txt".

```
<Project>
Title = "QOL Data";
Description = "Quality of life + ERS + MRS with sum-score to EAP translation for QOL";

<Options>
  Mode = Scoring;                              // Score responses.
```

```
  Score = SSC;                                    // Use sum score to scale score conversion.
  SaveSCO = Yes;                                  // Save scores to a file with "-sco.txt" extension.
  ReadPRMFile = "QOL_Calib_1dimERSMRS-prm.txt";   // For scoring, read item parameters from a saved "-prm.txt" file.

<Groups>

%Group1%
  File = "QOL.dat";           // The name of the data file that contains item responses.
  Varnames = y1-y35;          // Variable names for the data file. Short-hand is used to define y1 through y35.
  Ncats(y1-y35) = 7;          // Number of categories for variables y1-y35. Multiple Ncats statements are permitted.
  Model(y1-y35) = Nominal(7); // Item response model to use for variables y1-y35. Nominal model with 7 categories.
  Dimensions = 3;             // Number of latent dimensions. Here we have 3: QOL, ERS, MRS.
                              // Ta statement not necessary as "-prm.txt" file already contains Ta matrices used in estimation.

<Constraints> // No additional constraints necessary as item parameters already estimated.
```

Sum score to EAP translation: Extreme Response Style

The following contains line numbers for "QOL_SSEAP_ERS.txt".

```
1  <Project>
2  Title = "QOL Data";
3  Description = "1D + ERS + MRS with sum-score to EAP translation for ERS";
4
5  <Options>
6    Mode = Scoring;                                 // Score responses.
7    Score = SSC;                                    // Use sum score to scale score conversion.
8    SaveSCO = Yes;                                  // Save scores to a file with "-sco.txt" extension.
9    ReadPRMFile = "QOL_Calib_1dimERSMRS-prm.txt";   // For scoring, read item parameters from a saved "-prm.txt" file.
10
11 <Groups>
12
13 %Group1%
14   File = "QOL.dat";                          // The name of the data file that contains item responses.
15   Varnames = y1-y35;                         // Variable names for the data file. Short-hand is used to define y1 through y35.
16   Ncats(y1-y35) = 7;                         // Number of categories for variables y1-y35. Multiple Ncats statements are permitted.
17   Model(y1-y35) = Nominal(7);                // Item response model to use for variables y1-y35. Nominal model with 7 categories.
18   Dimensions = 3;                            // Number of latent dimensions. Here we have 3: QOL, ERS, MRS.
19   ItemWeights(y1-y35) = (1.0,0,0,0,0,0,1.0); // Item weights for sum score to scale score conversion for ERS.
20                                              // Ta statement not necessary as "-prm.txt" file already contains Ta matrices used in
                                                    estimation.
21 <Constraints> // No additional constraints necessary as item parameters already estimated.
```

2 *mirt*

The following code snippet for *mirt* (Chalmers, 2012) can be used to fit all four models that are discussed in the main text of the manuscript. The last line provides an example of how EAP scores can then be computed.

```
library(mirt)

dat<-read.table("QOL.dat")
dat<-dat[,1:35] # last column is an respondent index

# Fit GPC for just QOL
fit.gpc<-mirt(dat,1,itemtype="gpcm",technical=list(NCYCLES=1000))
fit.gpc


# Custom scoring functions for QOL and ERS
sf.qolers<-list()
for(i in 1:35){
  sf.qolers[[i]]<-matrix(
    c(
      0,1,2,3,4,5,6, # QOL
      1,0,0,0,0,0,1  # ERS
    ), 7, 2)
}

# Define model: all items load on both dimensions
# Estimate covariance between QOL and ERS
mod.qolers<-"
  QOL = 1-35
  ERS = 1-35
  COV = QOL*ERS
"

# Estimate QOL and ERS model
fit.qolers<-mirt(dat,mod.qolers,itemtype="gpcm",gpcm_mats=sf.qolers,technical=list(NCYCLES=1000))
fit.qolers


# Custom scoring functions for QOL and MRS
sf.qolmrs<-list()
for(i in 1:35){
  sf.qolmrs[[i]]<-matrix(
    c(
      0,1,2,3,4,5,6, # QOL
      0,0,0,1,0,0,0  # MRS
    ), 7, 2)
}

# Define model: all items load on both dimensions
# Estimate covariance between QOL and ERS
mod.qolmrs<-"
  QOL = 1-35
  MRS = 1-35
  COV = QOL*MRS
"

# Estimate QOL and MRS model
fit.qolmrs<-mirt(dat,mod.qolmrs,itemtype="gpcm",gpcm_mats=sf.qolmrs,technical=list(NCYCLES=1000))
fit.qolmrs


# Custom scoring functions for QOL, ERS, and MRS
sf.qolersmrs<-list()
for(i in 1:35){
  sf.qolersmrs[[i]]<-matrix(
    c(
```

```
      0,1,2,3,4,5,6, # QOL
      1,0,0,0,0,0,1, # ERS
      0,0,0,1,0,0,0  # MRS
    ), 7, 3)
}

# Define model: all items load on both dimensions
# Estimate covariance between QOL and ERS
mod.qolersmrs<-"
  QOL = 1-35
  ERS = 1-35
  MRS = 1-35
  COV = QOL*ERS*MRS
"

# Estimate QOL, ERS, and MRS model
# More quadrature points appear to be required to get a closer result between this model
# and that produced by flexMIRT. mirt will otherwise automatically reduce the number
# of quadrature points for numerical integration as latent dimensions are added.
# With more than 3 dimensions, you're probably better off using the MHRM algorithm to
# estimate the model.
fit.qolersmrs<-mirt(dat,mod.qolersmrs,itemtype="gpcm",
                    quadpts=25,gpcm_mats=sf.qolersmrs,technical=list(NCYCLES=5000))
fit.qolersmrs


# EAP scores can be obtained using the fscores() function
# For example,
eap.qolersmrs<-fscores(fit.qolersmrs,method="EAP",quadpts=25)
```

3 *Mplus*

We assume that many readers may already be familiar with *Mplus*, however, for the sake of clarity the following *Mplus* control file uses the same original data file as that used in the other analyses and the constraint section lists new variables defined such that one can see the connection between output and the MNRM parameterization used in the main text. Some short-cuts may be possible to shorten the length of control files and/or eliminate some sections of the `CONSTRAINTS` section. In addition, although we only present the contents of the "ersmrs_reverse.inp" file, analogous sections to other control files ("gpc_reverse.inp", "ers_reverse.inp", and "mrs_reverse.inp") appear separately.

First, lines 7 through 41 accomplish reverse-coding of the items, as discussed in the main text of the manuscript (see also Huggins-Manley & Algina, 2015). The same variables names (y1 through y35) are reused and declared as nominal items (see lines 43 through 47). Use of nominal items and `ESTIMATOR=ML` (line 50) will result in use of the estimation approach by Bock and Aitkin (1981).

Next, lines 54 through 89 set free all category slopes for the QOL factor. Note that each individual category and dimension has an associated slope, $\tilde{a}_{kd}$. For example, `y1#1` through `y1#6` refer to six categories for the first item, y_1. The seventh (and final) category slope is not available and is automatically set to zero. Labels are used (e.g., `(L1_6 - L1_1)`) to keep track of the fact that items have been reverse coded. Note that for the QOL dimension, the first category slopes are set free (not fixed, as is the *Mplus* default) by use of an asterisk.

For ERS, all items again load on this factor (lines 91 through 126). However, note that the five middle categories for each item have starting values of negative 1 (i.e., `*-1`).

Given how the scoring function for ERS is defined, $\begin{bmatrix} 0 & -1 & -1 & -1 & -1 & -1 & 0 \end{bmatrix}$, this effectively avoids factor reflection. The MRS factor then spans lines 128 to 163. In this case, the middle category slopes for all items has a starting value of 1, and the scoring function is still $\begin{bmatrix} 0 & 0 & 0 & 1 & 0 & 0 & 0 \end{bmatrix}$

The mean and variance-covariances among latent variables are declared in lines 165 through 171, and all intercept terms are set free and labeled in lines 173 through 207.

Finally, we discuss elements of the `CONSTRAINT` section. Lines 214 through 270 declare new variables that will be computed from existing model parameters. These labels are helpful to the extent that they allow proper bookkeeping of slopes and intercepts for each factor, without the user having to inspect individual category slopes and remember which refers to the overall item slope. For instance, QOL slopes are equal to category slopes with a "`_1`" suffix (see lines 383 through 390), MRS slopes are equal to the middle category slope (with a "`m_3`" suffix; see lines 408-415). Lines 417 through 661 force *Mplus* to print the intercept terms; we note that otherwise these will appear in the reverse order in output due to reverse coding. In addition, for the ERS factor, we must remember that due to the scoring function definition, any of the middle categories are equal to the overall slope for the ERS factor, but with the opposite sign (lines 392 through 406). The above parameter estimates will appear in the *Mplus* output, labeled "`New/Additional Parameters`". In this case, we use the naming convention that "`I1`" refers to the first item, "`A1`" refers to overall item slopes on the first latent dimension (here, QOL), "`A2`" to the overall slope on the second dimension (ERS), "`A3`" for the third dimension (MRS), and parameters with a "`C`" suffix refer to intercepts.

Of course, linear equality constraints are also necessary in order to fix the scoring functions for each dimension to the desired values. Lines 273 through 307 do this for the QOL dimension. See, for example, line 273. Category slopes `L1_6` through `L1_2` are set as a linear function of the category slope `L1_1`, and it may be helpful to refer again to the main text of the paper. For example, it is mentioned in the main text because of the scoring function definition ($\begin{bmatrix} 6 & 5 & 4 & 3 & 2 & 1 & 0 \end{bmatrix}$), that $\tilde{a}_{4,QOL} = 2\tilde{a}_{5,QOL}$. Since we have reverse coded items and also added appropriate labels, this translates to `L1_2 = 2*L1_1;` for the current control file (In light of reverse coding, it is important and tricky to keep track of how the categories are coded). Analogous constraints are required for the remaining categories. For ERS, note how category slopes 5 through 2 are set equal to the first category slope, and the 6th slope is fixed to zero (lines 310 through 344). Again, this is because the scoring function definition dictates that the middle 5 category slopes are equal to each other. For MRS, we only need to constrain all category slopes (except the middle category, or category 3 in the control file) equal to zero (lines 346 through 381).

```
1  TITLE:  QOL + ERS + MRS example with GPC constraints on MNRM
2
3  DATA: FILE = QOL.dat;
4
5  DEFINE:
6    ! reverse code all items
7    y1 = 7-y1;
8    y2 = 7-y2;
9    y3 = 7-y3;
10   y4 = 7-y4;
```

```
    y5= 7-y5;
    y6 = 7-y6;
    y7 = 7-y7;
    y8 = 7-y8;
    y9 = 7-y9;
    y10 = 7-y10;
    y11 = 7-y11;
    y12 = 7-y12;
    y13 = 7-y13;
    y14 = 7-y14;
    y15 = 7-y15;
    y16 = 7-y16;
    y17 = 7-y17;
    y18 = 7-y18;
    y19 = 7-y19;
    y20 = 7-y20;
    y21 = 7-y21;
    y22 = 7-y22;
    y23 = 7-y23;
    y24 = 7-y24;
    y25 = 7-y25;
    y26 = 7-y26;
    y27 = 7-y27;
    y28 = 7-y28;
    y29 = 7-y29;
    y30 = 7-y30;
    y31 = 7-y31;
    y32 = 7-y32;
    y33 = 7-y33;
    y34 = 7-y34;
    y35 = 7-y35;

VARIABLE:
NAMES=y1-y35;
USEVARIABLES = y1-y35;
NOMINAL = y1-y35;
MISSING = y1-y35 (-9);

ANALYSIS:
ESTIMATOR=ML;
PROCESSORS = 6;

MODEL:
QOL BY
    y1#1* y1#2 y1#3 y1#4 y1#5 y1#6 (L1_6 - L1_1)
    y2#1* y2#2 y2#3 y2#4 y2#5 y2#6 (L2_6 - L2_1)
    y3#1* y3#2 y3#3 y3#4 y3#5 y3#6 (L3_6 - L3_1)
    y4#1* y4#2 y4#3 y4#4 y4#5 y4#6 (L4_6 - L4_1)
    y5#1* y5#2 y5#3 y5#4 y5#5 y5#6 (L5_6 - L5_1)
    y6#1* y6#2 y6#3 y6#4 y6#5 y6#6 (L6_6 - L6_1)
    y7#1* y7#2 y7#3 y7#4 y7#5 y7#6 (L7_6 - L7_1)
    y8#1* y8#2 y8#3 y8#4 y8#5 y8#6 (L8_6 - L8_1)
    y9#1* y9#2 y9#3 y9#4 y9#5 y9#6 (L9_6 - L9_1)
    y10#1* y10#2 y10#3 y10#4 y10#5 y10#6 (L10_6 - L10_1)
    y11#1* y11#2 y11#3 y11#4 y11#5 y11#6 (L11_6 - L11_1)
    y12#1* y12#2 y12#3 y12#4 y12#5 y12#6 (L12_6 - L12_1)
    y13#1* y13#2 y13#3 y13#4 y13#5 y13#6 (L13_6 - L13_1)
    y14#1* y14#2 y14#3 y14#4 y14#5 y14#6 (L14_6 - L14_1)
    y15#1* y15#2 y15#3 y15#4 y15#5 y15#6 (L15_6 - L15_1)
    y16#1* y16#2 y16#3 y16#4 y16#5 y16#6 (L16_6 - L16_1)
    y17#1* y17#2 y17#3 y17#4 y17#5 y17#6 (L17_6 - L17_1)
    y18#1* y18#2 y18#3 y18#4 y18#5 y18#6 (L18_6 - L18_1)
    y19#1* y19#2 y19#3 y19#4 y19#5 y19#6 (L19_6 - L19_1)
    y20#1* y20#2 y20#3 y20#4 y20#5 y20#6 (L20_6 - L20_1)
    y21#1* y21#2 y21#3 y21#4 y21#5 y21#6 (L21_6 - L21_1)
    y22#1* y22#2 y22#3 y22#4 y22#5 y22#6 (L22_6 - L22_1)
    y23#1* y23#2 y23#3 y23#4 y23#5 y23#6 (L23_6 - L23_1)
    y24#1* y24#2 y24#3 y24#4 y24#5 y24#6 (L24_6 - L24_1)
```

```
     y25#1* y25#2 y25#3 y25#4 y25#5 y25#6 (L25_6 - L25_1)
     y26#1* y26#2 y26#3 y26#4 y26#5 y26#6 (L26_6 - L26_1)
     y27#1* y27#2 y27#3 y27#4 y27#5 y27#6 (L27_6 - L27_1)
     y28#1* y28#2 y28#3 y28#4 y28#5 y28#6 (L28_6 - L28_1)
     y29#1* y29#2 y29#3 y29#4 y29#5 y29#6 (L29_6 - L29_1)
     y30#1* y30#2 y30#3 y30#4 y30#5 y30#6 (L30_6 - L30_1)
     y31#1* y31#2 y31#3 y31#4 y31#5 y31#6 (L31_6 - L31_1)
     y32#1* y32#2 y32#3 y32#4 y32#5 y32#6 (L32_6 - L32_1)
     y33#1* y33#2 y33#3 y33#4 y33#5 y33#6 (L33_6 - L33_1)
     y34#1* y34#2 y34#3 y34#4 y34#5 y34#6 (L34_6 - L34_1)
     y35#1* y35#2 y35#3 y35#4 y35#5 y35#6 (L35_6 - L35_1);

ERS BY
     y1#1* y1#2*-1 y1#3*-1 y1#4*-1 y1#5*-1 y1#6*-1 (L1e_6 - L1e_1)
     y2#1* y2#2*-1 y2#3*-1 y2#4*-1 y2#5*-1 y2#6*-1 (L2e_6 - L2e_1)
     y3#1* y3#2*-1 y3#3*-1 y3#4*-1 y3#5*-1 y3#6*-1 (L3e_6 - L3e_1)
     y4#1* y4#2*-1 y4#3*-1 y4#4*-1 y4#5*-1 y4#6*-1 (L4e_6 - L4e_1)
     y5#1* y5#2*-1 y5#3*-1 y5#4*-1 y5#5*-1 y5#6*-1 (L5e_6 - L5e_1)
     y6#1* y6#2*-1 y6#3*-1 y6#4*-1 y6#5*-1 y6#6*-1 (L6e_6 - L6e_1)
     y7#1* y7#2*-1 y7#3*-1 y7#4*-1 y7#5*-1 y7#6*-1 (L7e_6 - L7e_1)
     y8#1* y8#2*-1 y8#3*-1 y8#4*-1 y8#5*-1 y8#6*-1 (L8e_6 - L8e_1)
     y9#1* y9#2*-1 y9#3*-1 y9#4*-1 y9#5*-1 y9#6*-1 (L9e_6 - L9e_1)
     y10#1* y10#2*-1 y10#3*-1 y10#4*-1 y10#5*-1 y10#6*-1 (L10e_6 - L10e_1)
     y11#1* y11#2*-1 y11#3*-1 y11#4*-1 y11#5*-1 y11#6*-1 (L11e_6 - L11e_1)
     y12#1* y12#2*-1 y12#3*-1 y12#4*-1 y12#5*-1 y12#6*-1 (L12e_6 - L12e_1)
     y13#1* y13#2*-1 y13#3*-1 y13#4*-1 y13#5*-1 y13#6*-1 (L13e_6 - L13e_1)
     y14#1* y14#2*-1 y14#3*-1 y14#4*-1 y14#5*-1 y14#6*-1 (L14e_6 - L14e_1)
     y15#1* y15#2*-1 y15#3*-1 y15#4*-1 y15#5*-1 y15#6*-1 (L15e_6 - L15e_1)
     y16#1* y16#2*-1 y16#3*-1 y16#4*-1 y16#5*-1 y16#6*-1 (L16e_6 - L16e_1)
     y17#1* y17#2*-1 y17#3*-1 y17#4*-1 y17#5*-1 y17#6*-1 (L17e_6 - L17e_1)
     y18#1* y18#2*-1 y18#3*-1 y18#4*-1 y18#5*-1 y18#6*-1 (L18e_6 - L18e_1)
     y19#1* y19#2*-1 y19#3*-1 y19#4*-1 y19#5*-1 y19#6*-1 (L19e_6 - L19e_1)
     y20#1* y20#2*-1 y20#3*-1 y20#4*-1 y20#5*-1 y20#6*-1 (L20e_6 - L20e_1)
     y21#1* y21#2*-1 y21#3*-1 y21#4*-1 y21#5*-1 y21#6*-1 (L21e_6 - L21e_1)
     y22#1* y22#2*-1 y22#3*-1 y22#4*-1 y22#5*-1 y22#6*-1 (L22e_6 - L22e_1)
     y23#1* y23#2*-1 y23#3*-1 y23#4*-1 y23#5*-1 y23#6*-1 (L23e_6 - L23e_1)
     y24#1* y24#2*-1 y24#3*-1 y24#4*-1 y24#5*-1 y24#6*-1 (L24e_6 - L24e_1)
     y25#1* y25#2*-1 y25#3*-1 y25#4*-1 y25#5*-1 y25#6*-1 (L25e_6 - L25e_1)
     y26#1* y26#2*-1 y26#3*-1 y26#4*-1 y26#5*-1 y26#6*-1 (L26e_6 - L26e_1)
     y27#1* y27#2*-1 y27#3*-1 y27#4*-1 y27#5*-1 y27#6*-1 (L27e_6 - L27e_1)
     y28#1* y28#2*-1 y28#3*-1 y28#4*-1 y28#5*-1 y28#6*-1 (L28e_6 - L28e_1)
     y29#1* y29#2*-1 y29#3*-1 y29#4*-1 y29#5*-1 y29#6*-1 (L29e_6 - L29e_1)
     y30#1* y30#2*-1 y30#3*-1 y30#4*-1 y30#5*-1 y30#6*-1 (L30e_6 - L30e_1)
     y31#1* y31#2*-1 y31#3*-1 y31#4*-1 y31#5*-1 y31#6*-1 (L31e_6 - L31e_1)
     y32#1* y32#2*-1 y32#3*-1 y32#4*-1 y32#5*-1 y32#6*-1 (L32e_6 - L32e_1)
     y33#1* y33#2*-1 y33#3*-1 y33#4*-1 y33#5*-1 y33#6*-1 (L33e_6 - L33e_1)
     y34#1* y34#2*-1 y34#3*-1 y34#4*-1 y34#5*-1 y34#6*-1 (L34e_6 - L34e_1)
     y35#1* y35#2*-1 y35#3*-1 y35#4*-1 y35#5*-1 y35#6*-1 (L35e_6 - L35e_1);

MRS BY
     y1#1* y1#2 y1#3*1 y1#4 y1#5 y1#6 (L1m_6 - L1m_1)
     y2#1* y2#2 y2#3*1 y2#4 y2#5 y2#6 (L2m_6 - L2m_1)
     y3#1* y3#2 y3#3*1 y3#4 y3#5 y3#6 (L3m_6 - L3m_1)
     y4#1* y4#2 y4#3*1 y4#4 y4#5 y4#6 (L4m_6 - L4m_1)
     y5#1* y5#2 y5#3*1 y5#4 y5#5 y5#6 (L5m_6 - L5m_1)
     y6#1* y6#2 y6#3*1 y6#4 y6#5 y6#6 (L6m_6 - L6m_1)
     y7#1* y7#2 y7#3*1 y7#4 y7#5 y7#6 (L7m_6 - L7m_1)
     y8#1* y8#2 y8#3*1 y8#4 y8#5 y8#6 (L8m_6 - L8m_1)
     y9#1* y9#2 y9#3 y9#4 y9#5 y9#6 (L9m_6 - L9m_1)
     y10#1* y10#2 y10#3*1 y10#4 y10#5 y10#6 (L10m_6 - L10m_1)
     y11#1* y11#2 y11#3*1 y11#4 y11#5 y11#6 (L11m_6 - L11m_1)
     y12#1* y12#2 y12#3*1 y12#4 y12#5 y12#6 (L12m_6 - L12m_1)
     y13#1* y13#2 y13#3*1 y13#4 y13#5 y13#6 (L13m_6 - L13m_1)
     y14#1* y14#2 y14#3*1 y14#4 y14#5 y14#6 (L14m_6 - L14m_1)
     y15#1* y15#2 y15#3*1 y15#4 y15#5 y15#6 (L15m_6 - L15m_1)
     y16#1* y16#2 y16#3*1 y16#4 y16#5 y16#6 (L16m_6 - L16m_1)
     y17#1* y17#2 y17#3*1 y17#4 y17#5 y17#6 (L17m_6 - L17m_1)
     y18#1* y18#2 y18#3*1 y18#4 y18#5 y18#6 (L18m_6 - L18m_1)
```

```
147       y19#1* y19#2 y19#3*1 y19#4 y19#5 y19#6 (L19m_6 - L19m_1)
148       y20#1* y20#2 y20#3*1 y20#4 y20#5 y20#6 (L20m_6 - L20m_1)
149       y21#1* y21#2 y21#3*1 y21#4 y21#5 y21#6 (L21m_6 - L21m_1)
150       y22#1* y22#2 y22#3*1 y22#4 y22#5 y22#6 (L22m_6 - L22m_1)
151       y23#1* y23#2 y23#3*1 y23#4 y23#5 y23#6 (L23m_6 - L23m_1)
152       y24#1* y24#2 y24#3*1 y24#4 y24#5 y24#6 (L24m_6 - L24m_1)
153       y25#1* y25#2 y25#3*1 y25#4 y25#5 y25#6 (L25m_6 - L25m_1)
154       y26#1* y26#2 y26#3*1 y26#4 y26#5 y26#6 (L26m_6 - L26m_1)
155       y27#1* y27#2 y27#3*1 y27#4 y27#5 y27#6 (L27m_6 - L27m_1)
156       y28#1* y28#2 y28#3*1 y28#4 y28#5 y28#6 (L28m_6 - L28m_1)
157       y29#1* y29#2 y29#3*1 y29#4 y29#5 y29#6 (L29m_6 - L29m_1)
158       y30#1* y30#2 y30#3*1 y30#4 y30#5 y30#6 (L30m_6 - L30m_1)
159       y31#1* y31#2 y31#3*1 y31#4 y31#5 y31#6 (L31m_6 - L31m_1)
160       y32#1* y32#2 y32#3*1 y32#4 y32#5 y32#6 (L32m_6 - L32m_1)
161       y33#1* y33#2 y33#3*1 y33#4 y33#5 y33#6 (L33m_6 - L33m_1)
162       y34#1* y34#2 y34#3*1 y34#4 y34#5 y34#6 (L34m_6 - L34m_1)
163       y35#1* y35#2 y35#3*1 y35#4 y35#5 y35#6 (L35m_6 - L35m_1);
164
165   QOL@1; !set the variance of QOL to be 1.
166   [QOL@0]; !set the mean of QOL to be 0.
167   ERS@1; [ERS@0];
168   MRS@1; [MRS@0];
169   QOL WITH MRS; !cov/cor between QOL and MRS.
170   QOL WITH ERS; !cov/cor between QOL and ERS.
171   MRS WITH ERS; !cov/cor between MRS and ERS.
172
173   [y1#1 y1#2 y1#3 y1#4 y1#5 y1#6] (I1_Int6 - I1_Int1);
174   [y2#1 y2#2 y2#3 y2#4 y2#5 y2#6] (I2_Int6 - I2_Int1);
175   [y3#1 y3#2 y3#3 y3#4 y3#5 y3#6] (I3_Int6 - I3_Int1);
176   [y4#1 y4#2 y4#3 y4#4 y4#5 y4#6] (I4_Int6 - I4_Int1);
177   [y5#1 y5#2 y5#3 y5#4 y5#5 y5#6] (I5_Int6 - I5_Int1);
178   [y6#1 y6#2 y6#3 y6#4 y6#5 y6#6] (I6_Int6 - I6_Int1);
179   [y7#1 y7#2 y7#3 y7#4 y7#5 y7#6] (I7_Int6 - I7_Int1);
180   [y8#1 y8#2 y8#3 y8#4 y8#5 y8#6] (I8_Int6 - I8_Int1);
181   [y9#1 y9#2 y9#3 y9#4 y9#5 y9#6] (I9_Int6 - I9_Int1);
182   [y10#1 y10#2 y10#3 y10#4 y10#5 y10#6] (I10_Int6 - I10_Int1);
183   [y11#1 y11#2 y11#3 y11#4 y11#5 y11#6] (I11_Int6 - I11_Int1);
184   [y12#1 y12#2 y12#3 y12#4 y12#5 y12#6] (I12_Int6 - I12_Int1);
185   [y13#1 y13#2 y13#3 y13#4 y13#5 y13#6] (I13_Int6 - I13_Int1);
186   [y14#1 y14#2 y14#3 y14#4 y14#5 y14#6] (I14_Int6 - I14_Int1);
187   [y15#1 y15#2 y15#3 y15#4 y15#5 y15#6] (I15_Int6 - I15_Int1);
188   [y16#1 y16#2 y16#3 y16#4 y16#5 y16#6] (I16_Int6 - I16_Int1);
189   [y17#1 y17#2 y17#3 y17#4 y17#5 y17#6] (I17_Int6 - I17_Int1);
190   [y18#1 y18#2 y18#3 y18#4 y18#5 y18#6] (I18_Int6 - I18_Int1);
191   [y19#1 y19#2 y19#3 y19#4 y19#5 y19#6] (I19_Int6 - I19_Int1);
192   [y20#1 y20#2 y20#3 y20#4 y20#5 y20#6] (I20_Int6 - I20_Int1);
193   [y21#1 y21#2 y21#3 y21#4 y21#5 y21#6] (I21_Int6 - I21_Int1);
194   [y22#1 y22#2 y22#3 y22#4 y22#5 y22#6] (I22_Int6 - I22_Int1);
195   [y23#1 y23#2 y23#3 y23#4 y23#5 y23#6] (I23_Int6 - I23_Int1);
196   [y24#1 y24#2 y24#3 y24#4 y24#5 y24#6] (I24_Int6 - I24_Int1);
197   [y25#1 y25#2 y25#3 y25#4 y25#5 y25#6] (I25_Int6 - I25_Int1);
198   [y26#1 y26#2 y26#3 y26#4 y26#5 y26#6] (I26_Int6 - I26_Int1);
199   [y27#1 y27#2 y27#3 y27#4 y27#5 y27#6] (I27_Int6 - I27_Int1);
200   [y28#1 y28#2 y28#3 y28#4 y28#5 y28#6] (I28_Int6 - I28_Int1);
201   [y29#1 y29#2 y29#3 y29#4 y29#5 y29#6] (I29_Int6 - I29_Int1);
202   [y30#1 y30#2 y30#3 y30#4 y30#5 y30#6] (I30_Int6 - I30_Int1);
203   [y31#1 y31#2 y31#3 y31#4 y31#5 y31#6] (I31_Int6 - I31_Int1);
204   [y32#1 y32#2 y32#3 y32#4 y32#5 y32#6] (I32_Int6 - I32_Int1);
205   [y33#1 y33#2 y33#3 y33#4 y33#5 y33#6] (I33_Int6 - I33_Int1);
206   [y34#1 y34#2 y34#3 y34#4 y34#5 y34#6] (I34_Int6 - I34_Int1);
207   [y35#1 y35#2 y35#3 y35#4 y35#5 y35#6] (I35_Int6 - I35_Int1);
208
209
210
211   Model CONSTRAINT:
212
213   ! Slopes
214   NEW (I1_a1 I2_a1 I3_a1 I4_a1 I5_a1 I6_a1
```

```
      I7_a1 I8_a1 I9_a1 I10_a1 I11_a1 I12_a1
      I13_a1 I14_a1 I15_a1 I16_a1 I17_a1 I18_a1
      I19_a1 I20_a1 I21_a1 I22_a1 I23_a1 I24_a1
      I25_a1 I26_a1 I27_a1 I28_a1 I29_a1 I30_a1
      I31_a1 I32_a1 I33_a1 I34_a1 I35_a1

      I1_a2 I2_a2 I3_a2 I4_a2 I5_a2 I6_a2
      I7_a2 I8_a2 I9_a2 I10_a2 I11_a2 I12_a2
      I13_a2 I14_a2 I15_a2 I16_a2 I17_a2 I18_a2
      I19_a2 I20_a2 I21_a2 I22_a2 I23_a2 I24_a2
      I25_a2 I26_a2 I27_a2 I28_a2 I29_a2 I30_a2
      I31_a2 I32_a2 I33_a2 I34_a2 I35_a2

      I1_a3 I2_a3 I3_a3 I4_a3 I5_a3 I6_a3
      I7_a3 I8_a3 I9_a3 I10_a3 I11_a3 I12_a3
      I13_a3 I14_a3 I15_a3 I16_a3 I17_a3 I18_a3
      I19_a3 I20_a3 I21_a3 I22_a3 I23_a3 I24_a3
      I25_a3 I26_a3 I27_a3 I28_a3 I29_a3 I30_a3
      I31_a3 I32_a3 I33_a3 I34_a3 I35_a3);

  ! Intercepts
  NEW (I1_c1 I1_c2 I1_c3 I1_c4 I1_c5 I1_c6
       I2_c1 I2_c2 I2_c3 I2_c4 I2_c5 I2_c6
       I3_c1 I3_c2 I3_c3 I3_c4 I3_c5 I3_c6
       I4_c1 I4_c2 I4_c3 I4_c4 I4_c5 I4_c6
       I5_c1 I5_c2 I5_c3 I5_c4 I5_c5 I5_c6
       I6_c1 I6_c2 I6_c3 I6_c4 I6_c5 I6_c6
       I7_c1 I7_c2 I7_c3 I7_c4 I7_c5 I7_c6
       I8_c1 I8_c2 I8_c3 I8_c4 I8_c5 I8_c6
       I9_c1 I9_c2 I9_c3 I9_c4 I9_c5 I9_c6
       I10_c1 I10_c2 I10_c3 I10_c4 I10_c5 I10_c6
       I11_c1 I11_c2 I11_c3 I11_c4 I11_c5 I11_c6
       I12_c1 I12_c2 I12_c3 I12_c4 I12_c5 I12_c6
       I13_c1 I13_c2 I13_c3 I13_c4 I13_c5 I13_c6
       I14_c1 I14_c2 I14_c3 I14_c4 I14_c5 I14_c6
       I15_c1 I15_c2 I15_c3 I15_c4 I15_c5 I15_c6
       I16_c1 I16_c2 I16_c3 I16_c4 I16_c5 I16_c6
       I17_c1 I17_c2 I17_c3 I17_c4 I17_c5 I17_c6
       I18_c1 I18_c2 I18_c3 I18_c4 I18_c5 I18_c6
       I19_c1 I19_c2 I19_c3 I19_c4 I19_c5 I19_c6
       I20_c1 I20_c2 I20_c3 I20_c4 I20_c5 I20_c6
       I21_c1 I21_c2 I21_c3 I21_c4 I21_c5 I21_c6
       I22_c1 I22_c2 I22_c3 I22_c4 I22_c5 I22_c6
       I23_c1 I23_c2 I23_c3 I23_c4 I23_c5 I23_c6
       I24_c1 I24_c2 I24_c3 I24_c4 I24_c5 I24_c6
       I25_c1 I25_c2 I25_c3 I25_c4 I25_c5 I25_c6
       I26_c1 I26_c2 I26_c3 I26_c4 I26_c5 I26_c6
       I27_c1 I27_c2 I27_c3 I27_c4 I27_c5 I27_c6
       I28_c1 I28_c2 I28_c3 I28_c4 I28_c5 I28_c6
       I29_c1 I29_c2 I29_c3 I29_c4 I29_c5 I29_c6
       I30_c1 I30_c2 I30_c3 I30_c4 I30_c5 I30_c6
       I31_c1 I31_c2 I31_c3 I31_c4 I31_c5 I31_c6
       I32_c1 I32_c2 I32_c3 I32_c4 I32_c5 I32_c6
       I33_c1 I33_c2 I33_c3 I33_c4 I33_c5 I33_c6
       I34_c1 I34_c2 I34_c3 I34_c4 I34_c5 I34_c6
       I35_c1 I35_c2 I35_c3 I35_c4 I35_c5 I35_c6);

  ! Constraints necessary for GPC slopes
  L1_6=6*L1_1; L1_5=5*L1_1; L1_4=4*L1_1; L1_3=3*L1_1; L1_2=2*L1_1;
  L2_6=6*L2_1; L2_5=5*L2_1; L2_4=4*L2_1; L2_3=3*L2_1; L2_2=2*L2_1;
  L3_6=6*L3_1; L3_5=5*L3_1; L3_4=4*L3_1; L3_3=3*L3_1; L3_2=2*L3_1;
  L4_6=6*L4_1; L4_5=5*L4_1; L4_4=4*L4_1; L4_3=3*L4_1; L4_2=2*L4_1;
  L5_6=6*L5_1; L5_5=5*L5_1; L5_4=4*L5_1; L5_3=3*L5_1; L5_2=2*L5_1;
  L6_6=6*L6_1; L6_5=5*L6_1; L6_4=4*L6_1; L6_3=3*L6_1; L6_2=2*L6_1;
  L7_6=6*L7_1; L7_5=5*L7_1; L7_4=4*L7_1; L7_3=3*L7_1; L7_2=2*L7_1;
  L8_6=6*L8_1; L8_5=5*L8_1; L8_4=4*L8_1; L8_3=3*L8_1; L8_2=2*L8_1;
  L9_6=6*L9_1; L9_5=5*L9_1; L9_4=4*L9_1; L9_3=3*L9_1; L9_2=2*L9_1;
  L10_6=6*L10_1; L10_5=5*L10_1; L10_4=4*L10_1; L10_3=3*L10_1; L10_2=2*L10_1;
```

```
L11_6=6*L11_1; L11_5=5*L11_1; L11_4=4*L11_1; L11_3=3*L11_1; L11_2=2*L11_1;
L12_6=6*L12_1; L12_5=5*L12_1; L12_4=4*L12_1; L12_3=3*L12_1; L12_2=2*L12_1;
L13_6=6*L13_1; L13_5=5*L13_1; L13_4=4*L13_1; L13_3=3*L13_1; L13_2=2*L13_1;
L14_6=6*L14_1; L14_5=5*L14_1; L14_4=4*L14_1; L14_3=3*L14_1; L14_2=2*L14_1;
L15_6=6*L15_1; L15_5=5*L15_1; L15_4=4*L15_1; L15_3=3*L15_1; L15_2=2*L15_1;
L16_6=6*L16_1; L16_5=5*L16_1; L16_4=4*L16_1; L16_3=3*L16_1; L16_2=2*L16_1;
L17_6=6*L17_1; L17_5=5*L17_1; L17_4=4*L17_1; L17_3=3*L17_1; L17_2=2*L17_1;
L18_6=6*L18_1; L18_5=5*L18_1; L18_4=4*L18_1; L18_3=3*L18_1; L18_2=2*L18_1;
L19_6=6*L19_1; L19_5=5*L19_1; L19_4=4*L19_1; L19_3=3*L19_1; L19_2=2*L19_1;
L20_6=6*L20_1; L20_5=5*L20_1; L20_4=4*L20_1; L20_3=3*L20_1; L20_2=2*L20_1;
L21_6=6*L21_1; L21_5=5*L21_1; L21_4=4*L21_1; L21_3=3*L21_1; L21_2=2*L21_1;
L22_6=6*L22_1; L22_5=5*L22_1; L22_4=4*L22_1; L22_3=3*L22_1; L22_2=2*L22_1;
L23_6=6*L23_1; L23_5=5*L23_1; L23_4=4*L23_1; L23_3=3*L23_1; L23_2=2*L23_1;
L24_6=6*L24_1; L24_5=5*L24_1; L24_4=4*L24_1; L24_3=3*L24_1; L24_2=2*L24_1;
L25_6=6*L25_1; L25_5=5*L25_1; L25_4=4*L25_1; L25_3=3*L25_1; L25_2=2*L25_1;
L26_6=6*L26_1; L26_5=5*L26_1; L26_4=4*L26_1; L26_3=3*L26_1; L26_2=2*L26_1;
L27_6=6*L27_1; L27_5=5*L27_1; L27_4=4*L27_1; L27_3=3*L27_1; L27_2=2*L27_1;
L28_6=6*L28_1; L28_5=5*L28_1; L28_4=4*L28_1; L28_3=3*L28_1; L28_2=2*L28_1;
L29_6=6*L29_1; L29_5=5*L29_1; L29_4=4*L29_1; L29_3=3*L29_1; L29_2=2*L29_1;
L30_6=6*L30_1; L30_5=5*L30_1; L30_4=4*L30_1; L30_3=3*L30_1; L30_2=2*L30_1;
L31_6=6*L31_1; L31_5=5*L31_1; L31_4=4*L31_1; L31_3=3*L31_1; L31_2=2*L31_1;
L32_6=6*L32_1; L32_5=5*L32_1; L32_4=4*L32_1; L32_3=3*L32_1; L32_2=2*L32_1;
L33_6=6*L33_1; L33_5=5*L33_1; L33_4=4*L33_1; L33_3=3*L33_1; L33_2=2*L33_1;
L34_6=6*L34_1; L34_5=5*L34_1; L34_4=4*L34_1; L34_3=3*L34_1; L34_2=2*L34_1;
L35_6=6*L35_1; L35_5=5*L35_1; L35_4=4*L35_1; L35_3=3*L35_1; L35_2=2*L35_1;

! Constraints necessary for ERS slopes
L1e_6=0; L1e_5=L1e_1; L1e_4=L1e_1; L1e_3=L1e_1; L1e_2=L1e_1;
L2e_6=0; L2e_5=L2e_1; L2e_4=L2e_1; L2e_3=L2e_1; L2e_2=L2e_1;
L3e_6=0; L3e_5=L3e_1; L3e_4=L3e_1; L3e_3=L3e_1; L3e_2=L3e_1;
L4e_6=0; L4e_5=L4e_1; L4e_4=L4e_1; L4e_3=L4e_1; L4e_2=L4e_1;
L5e_6=0; L5e_5=L5e_1; L5e_4=L5e_1; L5e_3=L5e_1; L5e_2=L5e_1;
L6e_6=0; L6e_5=L6e_1; L6e_4=L6e_1; L6e_3=L6e_1; L6e_2=L6e_1;
L7e_6=0; L7e_5=L7e_1; L7e_4=L7e_1; L7e_3=L7e_1; L7e_2=L7e_1;
L8e_6=0; L8e_5=L8e_1; L8e_4=L8e_1; L8e_3=L8e_1; L8e_2=L8e_1;
L9e_6=0; L9e_5=L9e_1; L9e_4=L9e_1; L9e_3=L9e_1; L9e_2=L9e_1;
L10e_6=0; L10e_5=L10e_1; L10e_4=L10e_1; L10e_3=L10e_1; L10e_2=L10e_1;
L11e_6=0; L11e_5=L11e_1; L11e_4=L11e_1; L11e_3=L11e_1; L11e_2=L11e_1;
L12e_6=0; L12e_5=L12e_1; L12e_4=L12e_1; L12e_3=L12e_1; L12e_2=L12e_1;
L13e_6=0; L13e_5=L13e_1; L13e_4=L13e_1; L13e_3=L13e_1; L13e_2=L13e_1;
L14e_6=0; L14e_5=L14e_1; L14e_4=L14e_1; L14e_3=L14e_1; L14e_2=L14e_1;
L15e_6=0; L15e_5=L15e_1; L15e_4=L15e_1; L15e_3=L15e_1; L15e_2=L15e_1;
L16e_6=0; L16e_5=L16e_1; L16e_4=L16e_1; L16e_3=L16e_1; L16e_2=L16e_1;
L17e_6=0; L17e_5=L17e_1; L17e_4=L17e_1; L17e_3=L17e_1; L17e_2=L17e_1;
L18e_6=0; L18e_5=L18e_1; L18e_4=L18e_1; L18e_3=L18e_1; L18e_2=L18e_1;
L19e_6=0; L19e_5=L19e_1; L19e_4=L19e_1; L19e_3=L19e_1; L19e_2=L19e_1;
L20e_6=0; L20e_5=L20e_1; L20e_4=L20e_1; L20e_3=L20e_1; L20e_2=L20e_1;
L21e_6=0; L21e_5=L21e_1; L21e_4=L21e_1; L21e_3=L21e_1; L21e_2=L21e_1;
L22e_6=0; L22e_5=L22e_1; L22e_4=L22e_1; L22e_3=L22e_1; L22e_2=L22e_1;
L23e_6=0; L23e_5=L23e_1; L23e_4=L23e_1; L23e_3=L23e_1; L23e_2=L23e_1;
L24e_6=0; L24e_5=L24e_1; L24e_4=L24e_1; L24e_3=L24e_1; L24e_2=L24e_1;
L25e_6=0; L25e_5=L25e_1; L25e_4=L25e_1; L25e_3=L25e_1; L25e_2=L25e_1;
L26e_6=0; L26e_5=L26e_1; L26e_4=L26e_1; L26e_3=L26e_1; L26e_2=L26e_1;
L27e_6=0; L27e_5=L27e_1; L27e_4=L27e_1; L27e_3=L27e_1; L27e_2=L27e_1;
L28e_6=0; L28e_5=L28e_1; L28e_4=L28e_1; L28e_3=L28e_1; L28e_2=L28e_1;
L29e_6=0; L29e_5=L29e_1; L29e_4=L29e_1; L29e_3=L29e_1; L29e_2=L29e_1;
L30e_6=0; L30e_5=L30e_1; L30e_4=L30e_1; L30e_3=L30e_1; L30e_2=L30e_1;
L31e_6=0; L31e_5=L31e_1; L31e_4=L31e_1; L31e_3=L31e_1; L31e_2=L31e_1;
L32e_6=0; L32e_5=L32e_1; L32e_4=L32e_1; L32e_3=L32e_1; L32e_2=L32e_1;
L33e_6=0; L33e_5=L33e_1; L33e_4=L33e_1; L33e_3=L33e_1; L33e_2=L33e_1;
L34e_6=0; L34e_5=L34e_1; L34e_4=L34e_1; L34e_3=L34e_1; L34e_2=L34e_1;
L35e_6=0; L35e_5=L35e_1; L35e_4=L35e_1; L35e_3=L35e_1; L35e_2=L35e_1;

! Constraints necessary for MRS slopes
L1m_6=0; L1m_5=0; L1m_4=0; L1m_2=0; L1m_1=0;
L2m_6=0; L2m_5=0; L2m_4=0; L2m_2=0; L2m_1=0;
L3m_6=0; L3m_5=0; L3m_4=0; L3m_2=0; L3m_1=0;
L4m_6=0; L4m_5=0; L4m_4=0; L4m_2=0; L4m_1=0;
```

```
L5m_6=0; L5m_5=0; L5m_4=0; L5m_2=0; L5m_1=0;
L6m_6=0; L6m_5=0; L6m_4=0; L6m_2=0; L6m_1=0;
L7m_6=0; L7m_5=0; L7m_4=0; L7m_2=0; L7m_1=0;
L8m_6=0; L8m_5=0; L8m_4=0; L8m_2=0; L8m_1=0;
L9m_6=0; L9m_5=0; L9m_4=0; L9m_2=0; L9m_1=0;
L10m_6=0; L10m_5=0; L10m_4=0; L10m_2=0; L10m_1=0;
L11m_6=0; L11m_5=0; L11m_4=0; L11m_2=0; L11m_1=0;
L12m_6=0; L12m_5=0; L12m_4=0; L12m_2=0; L12m_1=0;
L13m_6=0; L13m_5=0; L13m_4=0; L13m_2=0; L13m_1=0;
L14m_6=0; L14m_5=0; L14m_4=0; L14m_2=0; L14m_1=0;
L15m_6=0; L15m_5=0; L15m_4=0; L15m_2=0; L15m_1=0;
L16m_6=0; L16m_5=0; L16m_4=0; L16m_2=0; L16m_1=0;
L17m_6=0; L17m_5=0; L17m_4=0; L17m_2=0; L17m_1=0;
L18m_6=0; L18m_5=0; L18m_4=0; L18m_2=0; L18m_1=0;
L19m_6=0; L19m_5=0; L19m_4=0; L19m_2=0; L19m_1=0;
L20m_6=0; L20m_5=0; L20m_4=0; L20m_2=0; L20m_1=0;
L21m_6=0; L21m_5=0; L21m_4=0; L21m_2=0; L21m_1=0;
L22m_6=0; L22m_5=0; L22m_4=0; L22m_2=0; L22m_1=0;
L23m_6=0; L23m_5=0; L23m_4=0; L23m_2=0; L23m_1=0;
L24m_6=0; L24m_5=0; L24m_4=0; L24m_2=0; L24m_1=0;
L25m_6=0; L25m_5=0; L25m_4=0; L25m_2=0; L25m_1=0;
L26m_6=0; L26m_5=0; L26m_4=0; L26m_2=0; L26m_1=0;
L27m_6=0; L27m_5=0; L27m_4=0; L27m_2=0; L27m_1=0;
L28m_6=0; L28m_5=0; L28m_4=0; L28m_2=0; L28m_1=0;
L29m_6=0; L29m_5=0; L29m_4=0; L29m_2=0; L29m_1=0;
L30m_6=0; L30m_5=0; L30m_4=0; L30m_2=0; L30m_1=0;
L31m_6=0; L31m_5=0; L31m_4=0; L31m_2=0; L31m_1=0;
L32m_6=0; L32m_5=0; L32m_4=0; L32m_2=0; L32m_1=0;
L33m_6=0; L33m_5=0; L33m_4=0; L33m_2=0; L33m_1=0;
L34m_6=0; L34m_5=0; L34m_4=0; L34m_2=0; L34m_1=0;
L35m_6=0; L35m_5=0; L35m_4=0; L35m_2=0; L35m_1=0;

! QOL overall slope for item j (a1) = L(j)_1
I1_a1 = L1_1; I2_a1 = L2_1; I3_a1 = L3_1; I4_a1 = L4_1; I5_a1 = L5_1;
I6_a1 = L6_1; I7_a1 = L7_1; I8_a1 = L8_1; I9_a1 = L9_1; I10_a1 = L10_1;
I11_a1 = L11_1; I12_a1 = L12_1; I13_a1 = L13_1; I14_a1 = L14_1; I15_a1 = L15_1;
I16_a1 = L16_1; I17_a1 = L17_1; I18_a1 = L18_1; I19_a1 = L19_1; I20_a1 = L20_1;
I21_a1 = L21_1; I22_a1 = L22_1; I23_a1 = L23_1; I24_a1 = L24_1; I25_a1 = L25_1;
I26_a1 = L26_1; I27_a1 = L27_1; I28_a1 = L28_1; I29_a1 = L29_1; I30_a1 = L30_1;
I31_a1 = L31_1; I32_a1 = L32_1; I33_a1 = L33_1; I34_a1 = L34_1; I35_a1 = L35_1;

! ERS overall slope for item j (a1) = L(j)e_1
I1_a2 = -L1e_1; I2_a2 = -L2e_1; I3_a2 = -L3e_1;
I4_a2 = -L4e_1; I5_a2 = -L5e_1;
I6_a2 = -L6e_1; I7_a2 = -L7e_1; I8_a2 = -L8e_1;
I9_a2 = -L9e_1; I10_a2 = -L10e_1;
I11_a2 = -L11e_1; I12_a2 = -L12e_1; I13_a2 = -L13e_1;
I14_a2 = -L14e_1; I15_a2 = -L15e_1;
I16_a2 = -L16e_1; I17_a2 = -L17e_1; I18_a2 = -L18e_1;
I19_a2 = -L19e_1; I20_a2 = -L20e_1;
I21_a2 = -L21e_1; I22_a2 = -L22e_1; I23_a2 = -L23e_1;
I24_a2 = -L24e_1; I25_a2 = -L25e_1;
I26_a2 = -L26e_1; I27_a2 = -L27e_1; I28_a2 = -L28e_1;
I29_a2 = -L29e_1; I30_a2 = -L30e_1;
I31_a2 = -L31e_1; I32_a2 = -L32e_1; I33_a2 = -L33e_1;
I34_a2 = -L34e_1; I35_a2 = -L35e_1;

! MRS overall slope for item j (a1) = L(j)m_3
I1_a3 = L1m_3; I2_a3 = L2m_3; I3_a3 = L3m_3; I4_a3 = L4m_3; I5_a3 = L5m_3;
I6_a3 = L6m_3; I7_a3 = L7m_3; I8_a3 = L8m_3; I9_a3 = L9m_3; I10_a3 = L10m_3;
I11_a3 = L11m_3; I12_a3 = L12m_3; I13_a3 = L13m_3; I14_a3 = L14m_3; I15_a3 = L15m_3;
I16_a3 = L16m_3; I17_a3 = L17m_3; I18_a3 = L18m_3; I19_a3 = L19m_3; I20_a3 = L20m_3;
I21_a3 = L21m_3; I22_a3 = L22m_3; I23_a3 = L23m_3; I24_a3 = L24m_3; I25_a3 = L25m_3;
I26_a3 = L26m_3; I27_a3 = L27m_3; I28_a3 = L28m_3; I29_a3 = L29m_3; I30_a3 = L30m_3;
I31_a3 = L31m_3; I32_a3 = L32m_3; I33_a3 = L33m_3; I34_a3 = L34m_3; I35_a3 = L35m_3;

! Intercepts
I1_c1 = I1_Int1;
```

```
  I1_c2 = I1_Int2;
  I1_c3 = I1_Int3;
  I1_c4 = I1_Int4;
  I1_c5 = I1_Int5;
  I1_c6 = I1_Int6;

  I2_c1 = I2_Int1;
  I2_c2 = I2_Int2;
  I2_c3 = I2_Int3;
  I2_c4 = I2_Int4;
  I2_c5 = I2_Int5;
  I2_c6 = I2_Int6;

  I3_c1 = I3_Int1;
  I3_c2 = I3_Int2;
  I3_c3 = I3_Int3;
  I3_c4 = I3_Int4;
  I3_c5 = I3_Int5;
  I3_c6 = I3_Int6;

  I4_c1 = I4_Int1;
  I4_c2 = I4_Int2;
  I4_c3 = I4_Int3;
  I4_c4 = I4_Int4;
  I4_c5 = I4_Int5;
  I4_c6 = I4_Int6;

  I5_c1 = I5_Int1;
  I5_c2 = I5_Int2;
  I5_c3 = I5_Int3;
  I5_c4 = I5_Int4;
  I5_c5 = I5_Int5;
  I5_c6 = I5_Int6;

  I6_c1 = I6_Int1;
  I6_c2 = I6_Int2;
  I6_c3 = I6_Int3;
  I6_c4 = I6_Int4;
  I6_c5 = I6_Int5;
  I6_c6 = I6_Int6;

  I7_c1 = I7_Int1;
  I7_c2 = I7_Int2;
  I7_c3 = I7_Int3;
  I7_c4 = I7_Int4;
  I7_c5 = I7_Int5;
  I7_c6 = I7_Int6;

  I8_c1 = I8_Int1;
  I8_c2 = I8_Int2;
  I8_c3 = I8_Int3;
  I8_c4 = I8_Int4;
  I8_c5 = I8_Int5;
  I8_c6 = I8_Int6;

  I9_c1 = I9_Int1;
  I9_c2 = I9_Int2;
  I9_c3 = I9_Int3;
  I9_c4 = I9_Int4;
  I9_c5 = I9_Int5;
  I9_c6 = I9_Int6;

  I10_c1 = I10_Int1;
  I10_c2 = I10_Int2;
  I10_c3 = I10_Int3;
  I10_c4 = I10_Int4;
  I10_c5 = I10_Int5;
  I10_c6 = I10_Int6;
```

```
487
488   I11_c1 = I11_Int1;
489   I11_c2 = I11_Int2;
490   I11_c3 = I11_Int3;
491   I11_c4 = I11_Int4;
492   I11_c5 = I11_Int5;
493   I11_c6 = I11_Int6;
494
495   I12_c1 = I12_Int1;
496   I12_c2 = I12_Int2;
497   I12_c3 = I12_Int3;
498   I12_c4 = I12_Int4;
499   I12_c5 = I12_Int5;
500   I12_c6 = I12_Int6;
501
502   I13_c1 = I13_Int1;
503   I13_c2 = I13_Int2;
504   I13_c3 = I13_Int3;
505   I13_c4 = I13_Int4;
506   I13_c5 = I13_Int5;
507   I13_c6 = I13_Int6;
508
509   I14_c1 = I14_Int1;
510   I14_c2 = I14_Int2;
511   I14_c3 = I14_Int3;
512   I14_c4 = I14_Int4;
513   I14_c5 = I14_Int5;
514   I14_c6 = I14_Int6;
515
516   I15_c1 = I15_Int1;
517   I15_c2 = I15_Int2;
518   I15_c3 = I15_Int3;
519   I15_c4 = I15_Int4;
520   I15_c5 = I15_Int5;
521   I15_c6 = I15_Int6;
522
523   I16_c1 = I16_Int1;
524   I16_c2 = I16_Int2;
525   I16_c3 = I16_Int3;
526   I16_c4 = I16_Int4;
527   I16_c5 = I16_Int5;
528   I16_c6 = I16_Int6;
529
530   I17_c1 = I17_Int1;
531   I17_c2 = I17_Int2;
532   I17_c3 = I17_Int3;
533   I17_c4 = I17_Int4;
534   I17_c5 = I17_Int5;
535   I17_c6 = I17_Int6;
536
537   I18_c1 = I18_Int1;
538   I18_c2 = I18_Int2;
539   I18_c3 = I18_Int3;
540   I18_c4 = I18_Int4;
541   I18_c5 = I18_Int5;
542   I18_c6 = I18_Int6;
543
544   I19_c1 = I19_Int1;
545   I19_c2 = I19_Int2;
546   I19_c3 = I19_Int3;
547   I19_c4 = I19_Int4;
548   I19_c5 = I19_Int5;
549   I19_c6 = I19_Int6;
550
551   I20_c1 = I20_Int1;
552   I20_c2 = I20_Int2;
553   I20_c3 = I20_Int3;
554   I20_c4 = I20_Int4;
```

```
555   I20_c5 = I20_Int5;
556   I20_c6 = I20_Int6;
557
558   I21_c1 = I21_Int1;
559   I21_c2 = I21_Int2;
560   I21_c3 = I21_Int3;
561   I21_c4 = I21_Int4;
562   I21_c5 = I21_Int5;
563   I21_c6 = I21_Int6;
564
565   I22_c1 = I22_Int1;
566   I22_c2 = I22_Int2;
567   I22_c3 = I22_Int3;
568   I22_c4 = I22_Int4;
569   I22_c5 = I22_Int5;
570   I22_c6 = I22_Int6;
571
572   I23_c1 = I23_Int1;
573   I23_c2 = I23_Int2;
574   I23_c3 = I23_Int3;
575   I23_c4 = I23_Int4;
576   I23_c5 = I23_Int5;
577   I23_c6 = I23_Int6;
578
579   I24_c1 = I24_Int1;
580   I24_c2 = I24_Int2;
581   I24_c3 = I24_Int3;
582   I24_c4 = I24_Int4;
583   I24_c5 = I24_Int5;
584   I24_c6 = I24_Int6;
585
586   I25_c1 = I25_Int1;
587   I25_c2 = I25_Int2;
588   I25_c3 = I25_Int3;
589   I25_c4 = I25_Int4;
590   I25_c5 = I25_Int5;
591   I25_c6 = I25_Int6;
592
593   I26_c1 = I26_Int1;
594   I26_c2 = I26_Int2;
595   I26_c3 = I26_Int3;
596   I26_c4 = I26_Int4;
597   I26_c5 = I26_Int5;
598   I26_c6 = I26_Int6;
599
600   I27_c1 = I27_Int1;
601   I27_c2 = I27_Int2;
602   I27_c3 = I27_Int3;
603   I27_c4 = I27_Int4;
604   I27_c5 = I27_Int5;
605   I27_c6 = I27_Int6;
606
607   I28_c1 = I28_Int1;
608   I28_c2 = I28_Int2;
609   I28_c3 = I28_Int3;
610   I28_c4 = I28_Int4;
611   I28_c5 = I28_Int5;
612   I28_c6 = I28_Int6;
613
614   I29_c1 = I29_Int1;
615   I29_c2 = I29_Int2;
616   I29_c3 = I29_Int3;
617   I29_c4 = I29_Int4;
618   I29_c5 = I29_Int5;
619   I29_c6 = I29_Int6;
620
621   I30_c1 = I30_Int1;
622   I30_c2 = I30_Int2;
```

```
  I30_c3 = I30_Int3;
  I30_c4 = I30_Int4;
  I30_c5 = I30_Int5;
  I30_c6 = I30_Int6;

  I31_c1 = I31_Int1;
  I31_c2 = I31_Int2;
  I31_c3 = I31_Int3;
  I31_c4 = I31_Int4;
  I31_c5 = I31_Int5;
  I31_c6 = I31_Int6;

  I32_c1 = I32_Int1;
  I32_c2 = I32_Int2;
  I32_c3 = I32_Int3;
  I32_c4 = I32_Int4;
  I32_c5 = I32_Int5;
  I32_c6 = I32_Int6;

  I33_c1 = I33_Int1;
  I33_c2 = I33_Int2;
  I33_c3 = I33_Int3;
  I33_c4 = I33_Int4;
  I33_c5 = I33_Int5;
  I33_c6 = I33_Int6;

  I34_c1 = I34_Int1;
  I34_c2 = I34_Int2;
  I34_c3 = I34_Int3;
  I34_c4 = I34_Int4;
  I34_c5 = I34_Int5;
  I34_c6 = I34_Int6;

  I35_c1 = I35_Int1;
  I35_c2 = I35_Int2;
  I35_c3 = I35_Int3;
  I35_c4 = I35_Int4;
  I35_c5 = I35_Int5;
  I35_c6 = I35_Int6;
```

References

Bock, R. D., & Aitkin, M. (1981). Marginal maximum likelihood estimation of item parameters: Application of an EM algorithm. *Psychometrika*, *46*, 443–459.

Bolt, D. M., & Newton, J. R. (2011). Multiscale measurement of extreme response style. *Educational and Psychological Measurement*, *71*, 814-833.

Cai, L. (2017). flexMIRT version 3.51: Flexible multilevel multidimensional item analysis and test scoring [Computer software]. Chapel Hill, NC: Vector Psychometric Group.

Cai, L., & Hansen, M. (2013). Limited-information goodness-of-fit testing of hierarchical item factor models. *British Journal of Mathematical and Statistical Psychology*, *66*(2), 245-276. doi: 10.1111/j.2044-8317.2012.02050.x

Cai, L., & Monroe, S. (2013). Irt model fit evaluation from theory to practice: Progress and some unanswered questions. *Measurement: Interdisciplinary Research and Perspectives*, *11*(3), 102-106. doi: 10.1080/15366367.2013.835172

Chalmers, R. P. (2012). mirt: A multidimensional item response theory package for the R environment. *Journal of Statistical Software*, *48*(6), 1–29. Retrieved from `http://www.jstatsoft.org/v48/i06/`

Chen, W. H., & Thissen, D. (1997). Local dependence indices for item pairs using item response theory. *Journal of Educational and Behavioral Statistics*, *22*, 265–289.

Falk, C. F., & Cai, L. (2016). A flexible full-information approach to the modeling of response styles. *Psychological Methods*, *21*, 328–347.

Hansen, M., Cai, L., Stucky, B. D., Tucker, J. S., Shadel, W. G., & Edelen, M. O. (2014). Methodology for developing and evaluating the promis® smoking item banks. *Nicotine & Tobacco Research*, *16*, S175-S189. Retrieved from `http://dx.doi.org/10.1093/ntr/ntt123` doi: 10.1093/ntr/ntt123

Houts, C. R., & Cai, L. (2016). flexMIRT user's manual version 3.5: Flexible multilevel multidimensional item analysis and teset scoring [Computer software]. Chapel Hill, NC: Author

Huggins-Manley, A. C., & Algina, J. (2015). The partial credit model and generalized partial credit model as constrained nominal response models, with applications in M*plus*. *Structural Equation Modeling*, *22*, 308-318.

Muthén & Muthén. (2008). Mplus (Version 5.0) [Computer software]. Los Angeles, CA: Author.

Thissen, D., & Cai, L. (2016). Nominal categories models. In W. J. van der Linden (Ed.), *Handbook of modern item response theory, volume one: Models* (p. 51-73). Boca Raton: Chapman & Hall.
